# Ritual to relief: ethical frontiers in repurposing psychoactive substances

**DOI:** 10.1017/S1092852924002232

**Published:** 2025-12-03

**Authors:** Emma O’Leary, Seetal Dodd, Stephen Stahl

**Affiliations:** 1 IMPACT- The Institute for Mental and Physical Health and Clinical Translation, School of Medicine, Deakin University, Geelong, Australia; 2 Mental Health – Drug and Alcohol Services, Barwon Health, Geelong, Australia; 3 Centre for Youth Mental Health, University of Melbourne, Parkville, Australia; 4 Department of Psychiatry, University of California San Diego, San Diego, CA, USA

**Keywords:** Psychoactive, psychedelic, cannabis, stimulants, ethics

## Abstract

Psychoactive substances, known for their acute impact on perception and cognition, are gaining attention for their potential therapeutic applications. Many of these substances are plant-derived with deep-rooted histories of use in non-medical contexts, where they have been viewed as either tools for social cohesion or sources of discord, depending on cultural and societal contexts. This review explores psychoactive substances; psychedelics, cannabis, and stimulants, in a legitimized medical context, focusing on the ethical considerations shaping research and the regulatory and prescribing challenges involved in translating these compounds into viable clinical treatments. It highlights the diverse voices; Indigenous, philosophical, psychiatric, and using communities advocating for careful consideration of their broader implications. Key issues include navigating the blurred boundaries between therapeutic benefit and potential misuse, ensuring rigorous scientific methodologies, and addressing the sociopolitical factors shaping public perception and policy. The article emphasizes the need for evidence-based frameworks that balance innovation with patient safety and calls for approaches that recognize the social and commercial determinants of health, extending ethical considerations beyond merely prescribing. By critically assessing the promise and limitations of repurposing these substances, the article contributes to the ongoing discourse on their role in contemporary psychiatric practice.

## Introduction

Compared with other fields of medicine, drug discovery and development in psychiatry have been relatively underwhelming. The problems seem infinite. Disorders of the mind are highly variable. Symptomatology is wide-ranging and differs significantly between individuals. Classification strategies rely on subjective, imprecise methods. No powerfully singular pathophysiological pathways have been identified, rather inferences are made.

The central nervous system is also endlessly complex. Our understanding of the genetic and neural intricacies is embryonic. The brain generates sophisticated patterns of activity and responses that define its spatiotemporal organization. While this reorganizing capacity offers great promise it also leaves us vulnerable. Pathophysiological changes can be progressive and treatment remission remains a significant issue for the majority of psychiatric disorders.

Likewise, human behavior is complex and not neatly explained by the sum of its parts. Translating treatment into practice has been incredibly challenging. Pharmacotherapeutic research that traditionally relies on a receptor-ligand model is overly simplistic and does not account for the vast array of possibilities. With no cohesive way to explain disease states or the brain, we are working in the shadows.

There is however a growing urgency to uncover novel psychiatric treatments. Mental, neurological, and substance use disorders account for 10% of the global burden of disease, imposing significant economic costs, and are among the leading causes of disability. One in every hundred deaths is by suicide, with its impact reverberating through communities.^1^ Furthermore, climate change is expected to provoke both chronic and acute mental health conditions, amplifying the need for innovative solutions.

In this context, psychoactive substances, known for their acute, mind-altering capabilities are being increasingly investigated for their therapeutic potential. Many of these substances are plant-derived and have a long history of use in non-medical contexts, seen as either tools for social cohesion or sources of discord, depending on cultural and societal frameworks. In 18th-century Europe, there was a pivotal shift in thinking when the effects of these substances began to be understood less as spiritual or transcendent but rather as having physiological underpinnings.^2^ This narrative shift played a crucial role in an evolving understanding of psychoactive substances, which have since navigated a complex and circuitous route, alternatively classified as recreational or medical across different historical phases. With psychiatric disorders and the brain only partially understood, research has been increasingly turning to work in the shadows; revisiting these substances to explore their therapeutic potential, while also challenging the limitations and boundaries of traditional scientific and medical models.

Among these substances are psychedelics which in the broadest sense include 5-hydroxytryptamine receptor 2A (5-HT2A) agonists such as lysergic acid diethylamide (LSD), psilocybin and other dimethyltryptamine-containing substances, dissociative anesthetics like ketamine and scopolamine and various other hallucinogens such as ibogaine and salvia divinorium. The amphetamine-derived enactogen, 3,4-methylenedioxymethamphetamine (MDMA), produces some psychedelic-like effects but its hallucinogenic properties are less pronounced. These substances are being investigated as potential treatments for a wide range of conditions including treatment-resistant depression, post-traumatic stress disorder, cancer-related distress, and substance use disorders.

Concurrently, stimulants like amphetamines are increasingly prescribed for an expanding attention-deficit and hyperactivity disorder (ADHD) indication and we remain in the very active tail of the widespread opiate prescription experiment. At the risk of following a similar trajectory, cannabis medicines acting on the endocannabinoid system, are already in use for a wide range of conditions including chronic pain, anxiety, and sleep disorders despite a lack of robust evidence for some specific indications.

This unique ability to acutely alter the sensorium makes psychoactive substances differ from many established treatments. While a more integrative, nuanced approach is welcome, they demand a distinct kind of scrutiny. They can profoundly impact perception and consciousness and careful consideration of the risks and responsibilities is needed. Potential benefits must be carefully weighed against the risks of misuse, cultural extractionism, and environmental degradation, along with the unintended consequences of integrating these powerful agents into modern therapeutic practices.

Providing an exhaustive review of all the nuanced ethical issues facing this group of treatments is nearly impossible. While broader ethical issues apply to all psychiatric treatment and prescribing, this will focus on some of the central and contentious issues these newer pharmacotherapy approaches face, as well as referring to some of the generalities worth highlighting. It will be divided into three sections focusing on psychedelics, followed by cannabis medicines and stimulants with pertinent issues discussed in each group of treatments.

## Mystical medicine to clinical trials: navigating psychedelic dilemmas

In delving into the current landscape of psychedelic research and therapy, it is essential to confront the complex ethical challenges that accompany this resurgence. While commercial and sociopolitical efforts push to expand the availability of psychedelics, their legal status is in flux with varying degrees of acceptance and regulation across different regions. Indigenous, philosophical, psychiatric, and using communities are all calling for their voices to be heard as this process unfolds, emphasizing the need for careful consideration of the broader implications.

Psychedelics have a long history deeply embedded in the spiritual and cultural practices of diverse societies across time and space. From kykeon in the Eleusinian Mysteries of ancient Greece and soma in the Vedic rituals of the Brahmins, to amanita muscaria mushrooms in the cosmic visions of the Sami people of Sápmi, Lapland or the pituri plant in the Dreaming tracks on Mithaka Country, central Australia, to ayahuasca of the Shipibo people and Santo Daime practices in the Amazon Basin, or iboga in the Bwiti shrines of Gabon, these substances have been revered for their profound cultural and social significance, intertwined with rituals that hold deep meaning for those who use them.^3^

The profound impact of psychedelics on the mind, capable of dramatically altering perception and consciousness, adds another dimension of complexity. They can unearth deep psychological material, leading to intense interpersonal and existential challenges, making them susceptible to ethical misuse. This raises critical concerns about responsible administration, informed consent, and proper integration into therapeutic contexts. Moreover, the integration of mystical experiences into scientific paradigms remains contentious, often dismissed as unscientific, yet how central this is to their therapeutic potential remains unknown. Current scientific approaches also face challenges with study design issues, the difficulty of blinding, expectancy, and placebo effects, further complicating the scientific evaluation of these treatments.

Beyond the challenges common to this broader group of psychedelics, each drug presents its peculiar challenges. MDMA can lead to emotional openness and alterations in memory while ketamine presents unique risks and benefits, including rapid psychiatric symptom relief and dissociation that can complicate therapeutic relationships and long-term trajectories.

Cultural appropriation, cultural harm, and the impact of psychedelic tourism and neoshamanism on Indigenous communities are also pressing concerns, as is the potential for environmental degradation driven by increased demand for plant-derived psychedelics. While research in this field may reshape our understanding of consciousness and mental health, this too presents the need to consider how these powerful agents are integrated into modern therapeutic practices. This section aims to explore these issues in depth, acknowledging that while psychedelics hold immense promise, they also present a host of ethical challenges that must be navigated with care.

### Legal horizons: medical, cultural-spiritual, and emerging psychedelic contexts

In 2023, Australia was one of the first countries to make MDMA and psilocybin available for medical use with authorized psychiatrists becoming prescribers for the treatment of post-traumatic stress disorder and treatment-resistant depression respectively. While not yet approved in the United States (US) and Canada, special access schemes already permit the use of these treatments while they are still under investigation. Similarly, Switzerland allows the use of psychedelics in exceptional circumstances. There are also legislative moves to roll out ibogaine in Colorado and a scheme has already been implemented for psilocybin in Oregon. Meanwhile, ketamine is similarly being used to treat psychiatric conditions in Canada, the US, the UK, Australia, Germany, Spain, France, and the Czech Republic although some of this prescribing is off-label.

The cultural and religious uses of psychedelics continue to be recognized in certain regions, notably ayahuasca in most countries within the Amazon Basin, while Mexico holds special protection for peyote use by the Huichol people. Psychedelic tourism has also grown in these regions, as well as in Portugal, the Netherlands, and Costa Rica, where legal frameworks are either relaxed or ambiguous. The US, Canada, and New Zealand have all introduced specific legislation governing the cultural and religious use of psychedelics, allowing their use in specific community contexts. Meanwhile, over the past two decades, laws governing the recreational use of psychedelics have gradually relaxed in several countries, bringing these substances out of the shadows.

### Mind, molecule, and the echoes of the past: psychedelic psychopharmacology

The prevailing understanding of conventional therapeutics is that they induce minimal neuroplasticity initially, but gradually promote adaptive processes over weeks to months, generally leading to beneficial outcomes. In contrast, psychoactive substances produce effects relatively quickly. The long-term neurobiological changes associated with fast-acting but dependence-inducing substances are often less favorable. Such changes include altered reward circuitry, impaired neurogenesis, neuroinflammation, and neurotransmitter dysregulation. Psychedelics, however, stand apart. They can trigger rapid and profound neuropsychiatric changes after just one or a few doses, with effects that persist well beyond the substance’s presence in the body, and induced dependence is rare.^4^ This new paradigm casts a shadow over their use. While there is strong evidence of their therapeutic potential, the swift and lasting nature of these changes raises concerns about the neurobiological processes being altered in the long term. If not used carefully and knowledgeably, there is a significant risk of misuse, which could lead to serious harm.

There are various mechanisms by which psychedelics, especially psilocybin, are thought to act. None of which are mutually exclusive. Imaging studies show that a shift in neural connectivity appears to be at play. There are consistent findings of reduced activity in the default mode network (DMN), which is central to metacognition including self-referential processing. Correspondingly, there is an observable increase in global connectivity.^5^^,^^6^ Perturbations in DMN activity have been associated with many neuropsychiatric disorders, hence psychedelic therapy may act as a cognitive reset.

Mystical experiences and increased mental flexibility are conventionally thought to be key to their psychological mechanism and associated psychotherapeutic interventions.^7^ The intensity of mystical experiences has been found to correlate with the therapeutic response.^8^^–^^11^ High-dose psychedelics reliably induce both mystical-type experiences and hallucinations and patients often attribute benefit to an emotional breakthrough achieved while in an altered state of consciousness. But the legacy is longer than that. Psychedelics have long been valued for their capacity to conjure spiritual and mystical experiences in a variety of cultural and religious contexts. The recognition of this effect was foundational to early psychedelic research in the 1950s and 60s. Walter Pahnke’s 1962 landmark ‘Good Friday Experiment,’ held at a Protestant church service in Boston, showed profound mystical experiences could be induced across most participants in the active arm of the trial.^12^ This experiment bridged mystical traditions and scientific inquiry, paving the way for future psychedelic research.

Another explanation for the effect of psychedelics is neuroplasticity induced at a cellular and molecular level. It has been demonstrated that serotonergic psychedelics and ketamine bind at 5-HT2A receptors. These receptors have the greatest density in layers 4 and 5 of the cortical pyramidal neurons which play a central role in cognitive and processing capabilities including integrating sensory and subcortical information.^13^ After receptor binding, psychedelics induce a complex array of actions including via guanine nucleotide-binding proteins and arrestins, and rapidly promote synaptogenesis and dendritic arborization.^14^^,^^15^ Moving from subjective experiences to measurable biological processes has the potential to reduce the historical stigma that has hindered research progress.

The observed neuroplastic changes along with immunomodulation may address a pathway common to many psychiatric conditions characterized by neuroinflammation and a loss of neuroplasticity causing neural circuit dysfunction.^16^^,^^17^ Factors such as genetic vulnerabilities, childhood adversity, chronic stress and illness, and a disruption of the microbiome have all been shown to lead to a proinflammatory state resulting in a loss of synapses, dendritic arborizations, and poor synaptic maintenance.^15^^,^^18^ Furthermore, a handful of preclinical trials have established a relationship between these biological effects of psychedelics and adaptive behaviors.^13^ By targeting core neurobiological mechanisms, these findings hold the potential to move beyond symptom management, offering sustained recovery and even prevention. However, with all powerful treatments, the shadow of potential side effects looms, reminding us that these profound changes in the brain could also carry unintended consequences that must be carefully navigated.

Psychedelics and MDMA have actions beyond 5-HT2A receptors with LSD for example having a high binding affinity for most serotonin, noradrenaline, and dopamine receptors.^19^ Along with most psychedelics, MDMA has a high affinity for 5-HT2B and this binding site is problematic. With a high affinity for 5-HT2B, the repurposed antiepileptic medication, fenfluramine was withdrawn from the market in 1997 for its propensity to cause valvulopathies.^20^ Anoretic and ergot-derived drugs such as Parkinson’s medications also bind to 5-HT2B receptors causing the same serious side effect.^21^ Similarly, long-term MDMA use is associated with valvular heart disease.^20^^,^^22^

The cardiotoxicity of tryptamine-derived, ibogaine is more widely known and has been a major hurdle in its development as an addiction treatment. It modifies addiction-related neural pathways by upregulating neurotrophic factor signaling and open-label studies have shown a persistent reduction in drug-related behaviors after a single dose.^23^^–^^26^ Its cardiotoxic and arrhythmogenic potential is mediated through hERG potassium blockade.^27^^,^^28^ While there are no studies looking at the long-term cardiac side effects of psychedelics, repeat dosing including microdosing of all psychedelics should be considered with caution. Still much is not known about the human receptor pharmacology of psychedelics or of the drug–drug interactions with common psychotropic medications and both areas will require rigorous investigation for safe prescribing.

It is possible that the subjective experience of these psychoactive substances is simply undesirable, drug-induced side effects with non-hallucinogenic alternatives under investigation.^29^ Arguably, decoupling the beneficial effects from the hallucinogenic experience may reduce unpleasant and onerous side effects and potentially reduce risk, and cost, and improve user convenience. Microdosing is being explored. Preclinical work using animal models shows promise with the enantiomer, R-ketamine, tryptamine-derived AAZ, and ibogaine analogue tabernanthalog being investigated for the treatment of depression as well as opioid and alcohol dependence.^30^^–^^32^ While the subjective experience has been portrayed as delivering the greatest psychological insights, proponents of non-hallucinogenic psychedelics argue that the cellular effects may be adequate to achieve effective treatment and that psychotherapeutic catharsis associated with mystical experiences may merely reflect superfluous epiphenomenon of a biological mechanism such as 5-HT2A activation.^31^

Certainly, piggy-backing pharmaceutical discovery off traditionally used molecules is not unproblematic. Furthermore, these arguments risk oversimplifying complex human experiences by reducing them to mechanized biological processes, diminishing the rich cultural and psychosocial legacy of psychedelics. This approach can also perpetuate a form of cultural colonialism, appropriating what is perceived as useful while discarding what is uncomfortable or lacks a logical explanation. In tradition and in living cultural and religious practices, psychedelics are deeply embedded in systems where ritual and interpersonal bonding are integral to the healing process. Moreover, the scientific perspective, which sometimes acts as its own form of religion by prioritizing empirical evidence over other worldviews, may cast a shadow over the full therapeutic potential of these substances. While refining therapeutic molecules and reducing side effects is valid, rather than merely viewing psychedelics as tools for mechanistic intervention, they could present an opportunity to bridge psychotherapy and pharmacotherapeutic approaches, honoring both the experiential and the biological in the healing process.

### Trials and tribulations: scientific rigor in psychedelic methodology

One of the key issues with the available research is the generalizability of results. Most studies have small sample sizes and carefully selected subjects that are not necessarily representative of the clinical demographic where comorbid psychiatric conditions, medical illness, substance use, and polypharmacy are common.

Secondly, the clinical environment itself is carefully curated with many trials incorporating a rigorously structured, intensive therapeutic component, the replication of which in a non-research setting will be challenging and costly. The extent to which any putative benefits are due to the medication, the therapy, or the interaction has not been properly disentangled. This will have clinical and ethical implications for translation into clinical care. Therapists with less experience, less skill, or less knowledge may incur a different outcome to the overall treatment. While the structure of psychotherapy across trials has been fairly consistent involving preparation, medication support, and integration sessions, the therapy itself varies from trial to trial in the number of sessions provided and the therapeutic approach. Trials typically incorporate supportive or more directive approaches that are either non-intrusive or explicit and adapted from established modalities such as cognitive-behavioral, psychodynamic, and mindfulness-based approaches with some incorporating somatic therapies.^33^ What is unknown is the extent to which therapy plays a critical role and what type of therapy is best suited to which condition with current practice based on conventional wisdom.

Participant selection in psychedelic clinical trials is also an issue with Caucasian participants constituting over 80% of pooled analysis.^34^^,^^35^ Strategies that address this disparity are important for the generalizability of results and for addressing systemic issues more broadly. Racial trauma is significantly associated with worse mental health outcomes and Black, Indigenous, and People of Color, as well as LGBTQIA2+ individuals are disparately affected by the social determinants of health.^36^ The absence of this diversity will also affect the clinical applicability of findings.

Psychedelic research also faces some unique methodological challenges. Trials are vulnerable to selection bias and expectancy effects when treatment-experienced participants are included. Similar biases apply to participating therapists. One systematic review of trials of classical psychedelics found of the 4 out of 8 trials reporting on this data point, the majority of participants had used psychedelics before.^37^ Unsurprisingly, individuals who have used psychedelics before and had a positive experience would be more inclined to enroll. They are much more likely to recognize the effects of the drugs which would unblind the results and lead to positive expectancy and a potentially, inflated assessed benefit. They may also differ in temperament and other neuropsychological parameters such as harm avoidance and self-directedness.^38^ Previous use has also been shown to alter the acute effects and may also alter findings when it comes to psychological side effects.^39^ Sensational media portrayal and cultural enthusiasm could also influence the treatment of naïve participants, altering their expectations and artificially inflating positive findings.

Blinding in psychedelic randomized control trials (RCT) is particularly challenging due to the nature and intensity of alterations in mood, consciousness, and sensorium. Active placebos are often used in psychedelic drug trials but these do not constitute ideal alternatives. The symptoms induced by active placebos tend to be milder and they have different psychoactive profiles. They can induce potentially unpleasant side effects such as anxiety or dysphoria in the case of stimulants or be calmative in the case of sedatives and hypnotics like midazolam. Furthermore, information detailing the effects of psychedelics provided to trial participants while necessary, can also reduce masking. For all these factors, participants and investigators tend to reliably predict treatment allocation.^40^ An alternative strategy has been to use low dose or ascending doses of the trial drug but this eliminates the possibility of finding causal inference. Additionally, the patient-therapist alliance is likely to be altered if treatment unmasking occurs and this may positively skew findings.^41^ Experimental tools like the mystical experiences questionnaire 30, can also influence the subject’s interpretation of their experience.^41^^,^^42^ A particular concern pertains to the acute euphoric effects of psychedelics. In participants who have had persistent dysphoria and have been refractory to therapy, an acute and dramatic increase in mood would provide considerable hope and optimism which itself would have mood elevating and sustaining properties. While a cause for study design concern, perhaps it could also be a driver of benefit.

In a series of systematic reviews looking at expectancy, none of the 43 included studies measured participant pre-treatment expectancy, and only 5 of 30 ketamine for depression studies measured masking.^40^ Muthu Kumaraswamy et al. (2021) argue that blinding, expectancy, and patient-therapist alliance should be routinely measured in RCTs. There are also some suggestions that the use of mild deception or incomplete disclosure may be helpful in masking; for example, not disclosing the number of trial arms or getting participants to consent to a number of different psychoactive treatment arms when only two make up the trial. These strategies are not foolproof and also raise ethical questions.

An alternative means of addressing the expectancy effect is drug trials under anesthesia. One such study looking at the antidepressant effect of ketamine found no difference between the drug and control group. Masking was successful with 37% of participants correctly guessing their assigned arm.^43^ If the subjective experience is necessary for benefit or if therapy itself is synergistic, this study design eliminates those components. Furthermore, anesthetics can have an antidepressant effect and may also interfere with the mechanism of action of psychedelics.^44^

The external environment is well known to augment and potentiate the effects of psychedelics. While not unique to these psychoactive substances, the influence of extra-drug parameters appears to be exaggerated in their case.^45^^,^^46^ In recognition of this, set and setting form a key part of psychedelic clinical trials in an attempt to achieve neutrality and harm reduction. It is a historical legacy of psychedelic research and also carries a cultural context for using communities. More broadly, the sociocultural milieu in which drug trials are undertaken likely impacts their potential effects. Untampered claims or exaggeration of the benefits of psychedelics and uncritical hype may magnify expectancy effects. This is extremely important considering that treatment effect and expectancy likely operate synergistically via neurobiological mechanisms, according to early evidence.^44^

### Navigating consent: ethical imperatives in psychedelic-assisted therapy

Given the peculiarities of psychedelics, many have argued for augmented and standardized consent processes and protocols.^47^^,^^48^ As psychedelic therapies have the potential to present challenging experiences, induce personality changes, and call into question or alter an individual’s values and beliefs, adequate education on these possible occurrences is paramount.

Therapeutic touch, already debated in traditional psychotherapies, demands even stricter standards of practice and oversight in the psychedelic context. Heightened sexual, emotional, and physical sensitivity is especially common with MDMA.^49^ Current trial protocols require practitioners to work in pairs and only use touch that is explicitly requested by the participant. However, impaired autonomy and decision-making during psychedelic sessions along with the dynamic nature of consent complicates this practice. Challenges arise when a patient who initially declined touch changes their mind mid-session or initiates touch beyond the practitioner’s comfort or scope of practice. Omission of touch is also fraught, with some arguing it could limit therapeutic results and ad hoc capacity assessments may be countertherapeutic.^50^ Touch is particularly complicated given the high proportion of people with resistant depression and post-traumatic syndromes who have been the victims of prior sexual abuse or trauma –people disproportionately likely to receive such therapies.

Furthermore, a patient experiencing transient distress may wish to withdraw consent during an irreversible process. Thorough education, safety planning, and pre-session negotiations are critical. The definition of therapeutic touch is variable and this poses a risk of boundary transgression. Further complicating the picture are discourses on consent that argue it is not a simply binary but rather ambiguous and imbued with social relations of power.^51^^–^^53^ Clinical research on informed consent in those affected by trauma is limited.^54^ While adherence to pre-dosing agreements is crucial, developing dynamic protocols based on participant preferences and experiences and harnessing the wisdom of ethical frameworks used in nonmedical contexts should prove fruitful.^47^

The propensity of psychedelics to increase suggestibility may be key to their therapeutic potential in promoting social affiliation and mental flexibility. This effect, however, has also been shown to make individuals more vulnerable to persuasion, manipulation, and psychological destabilization. Historically, this led to lines of inquiry for the purposes of warfare and political gain. There are recent and historical cases involving sexual transgressions and the exploitation of participants in both legitimized and underground settings. There is a risk that awe induced by the subjective experience of the psychedelic may be confused with awe for the therapist. Given that these treatments may disproportionately be provided to vulnerable individuals impacted by trauma, safeguards will be essential. Ethical frameworks have been proposed to safeguard participants from potential harm.^55^ Additionally, the consequences of ethical breaches and procedures for addressing complaints should be clearly delineated and standardized.

Other factors may contribute to boundary transgressions. The nature of psychedelic interventions differs from the traditional training medical practitioners receive. These treatments are often viewed and portrayed as radical interventions, where new rules apply. This increases the potential for power imbalances and acts of coercion. In altered states, where ambiguity is heightened, it is imperative to maintain an acute awareness of the ethical risks, adhere to strict standards, and ensure practitioner integrity, adequate training, and oversight.

### Beyond the veil: mysticism, diversity, and the delicate dance of delusion

An area warranting closer examination is the terminology and theoretical framework of these treatments, where language and concepts can create ambiguity. The description and study of mystical-type experiences are prevalent in psychedelic literature. The legacy of this terminology draws from anthropology, religious and spiritual studies, and qualities intrinsic to the psychedelic experience itself with descriptors such as noetic and ineffable commonly used.^12^ As pointed out by Sanders et al. (2021), the problem arises when science emphasizes the therapeutic potency of the mystical experience. To the layperson, this could easily be conflated with an emphasis on the supernatural or divine.^56^ They argue that scrutiny of both the underlying psychobiological mechanisms and the theoretical frameworks for conscious states is needed to improve the translational potential of research and optimize therapeutic outcomes.

Others argue that this approach is reductionist and materialist.^57^ The concern being that narrowing the focus to not only Western ontological constructs but also to WEIRD (Western, Educated, Industrialized, Rich, and Democratic) subjects in combination with commercialization and medicalization of treatment, disadvantages diverse communities and devalues diverse meaning-making.^57^ Furthermore, they argue that while mystical descriptors may connote a non-naturalistic position, their framework remains agnostic.

Letheby et al. raise the important question, “How much, if at all, does it matter if psychedelic experiences involve real insights or ‘comforting delusions’?”^58^ From the standpoint of ethical pragmatism, one might argue that the therapeutic benefits could justify such experiences, even if they are not rooted in objective truth. However, this perspective invites further reflection on the implications of endorsing frameworks that may inadvertently privilege certain cultural or ontological constructs over others. The challenge, then, lies in balancing the need for rigorous scientific inquiry with respect for the diverse ways in which different communities understand and engage with these experiences. As the field continues to evolve it will be crucial to ensure that the frameworks used do not marginalize or disenfranchise those whose worldviews and experiences fall outside the dominant paradigms, thereby preserving the richness and diversity of meaning that psychedelic experiences can offer.

### Psychedelic tourism, neoshamanism and extractionism

While these treatments may be leaping from stigma to medicine, communities, particularly the Indigenous communities from which many of these substances were extracted, are overlooked. Indigenous communities have been responsible for conservation and sustainability practices along with cultural and ethical stewardship of land and plants, including psychoactive substances for thousands of years.^59^^,^^60^ Traditional knowledge and practices are increasingly being rightly recognized as having their own scientific validity and have been revolutionary in their impact on scientific thinking as well as thinking in other disciplines such as philosophy.^61^ Alternatives to Western paradigms and methodologies have radicalized concepts and practices including in the areas of bioconservation, physics, and medicine. Now is an opportunity to reflect such that the so-called psychedelic renaissance does not incur similar imperialistic harms as those incurred in the European Renaissance, which coincided with the plundering of the many colonized regions of the world.^61^ The legacy of this era and its sequelae, lived out in unjust and discriminatory practices and policies, continues to be felt and wreak intergenerational havoc. Furthermore, this historical legacy, along with further insults and desecrations is recognized as a significant social determinant of mental health.^59^

Many people are also traveling to countries with less restriction on psychedelics to access these substances for the purposes of spiritual and personal growth, healing, and cultural exploration. However, with this move comes inherent risks, not only in relation to safety, therapeutic effectiveness, adequate integration support, and exploitation by unscrupulous practitioners, but also regarding cultural and environmental harms. Concerns about the impact of tourism on culturally sacred substances like ayahuasca and peyote have prompted increased local regulation. Additionally, the commercialization and trivialization of cultural practices, along with the disruption of local resources and traditional ways of life, pose significant challenges. Some communities may struggle to maintain the cultural continuity of their practices in the face of these international demands.

Neoshamanism, which draws from Indigenous shamanic practices, raises several ethical concerns. These issues arise from the appropriation and reinterpretation of traditional practices by outsiders, often without the deep cultural, historical, and spiritual context in which they originated. Despite the therapeutic potential of psychedelics in non-medical contexts, neoshamanistic practices face ethical challenges, including a lack of regulation, the risk of exploiting and commodifying Indigenous traditions for profit and the potential to decenter, trivialize, and misrepresent these practices. Removing the cultural context may also remove established wisdom that forms a moral and therapeutic framework, which could safeguard participants’ well-being. Superficial and unsanctioned adoption of practices can falsely inflate a psychedelic guide’s legitimacy, leading to harm for participants in unregulated environments where they may fear speaking out, particularly if they face legal consequences for participating. Such practices may not only be seen as disrespectful but also highlight the need to recognize Indigenous sovereignty and ensure reciprocity with the communities from which these traditions originate.

The prohibitive cost of psychedelic treatments in a medical context is a significant concern, particularly regarding accessibility and equity. When these treatments become inaccessible due to high costs they risk being driven underground, where unregulated and unsafe practices may proliferate. The use of impure and contaminated substances, improper dosing, and a lack of skilled guidance, significantly increases the potential for harm. Vulnerable populations, unable to afford legal medical access are likely to be most affected, exacerbating existing health disparities. The ethical dilemma lies in balancing safety, public education, and availability, ensuring that access is not restricted to the wealthy while also preventing dangerous, illicit use due to cost barriers. The challenge for policymakers and the medical community is to create affordable, equitable pathways to treatment.

While predominating in nonmedical contexts, the interface between psychedelics and group settings has received little attention in the recent scientific resurgence.^62^ Given that psychotherapy contributes significantly to the expense, there is a pressing need for innovative approaches and research into new treatment paradigms to reduce costs while maintaining safety and efficacy. Without these efforts, the shadow of inaccessibility may undermine the promise of psychedelic medicine.

Indigenous people have a right to share in the profits and the benefits of psychoactive substances. They have a right to access the benefits of their cultural heritage and spiritual practices. Medical policies, while cautiously framing these substances to ensure public safety, also risk making them inaccessible to disadvantaged communities. Current research practices require multiple face-to-face individual sessions and the presence of two clinicians for dosing. This approach is costly. If these treatments have a place in modern psychiatry, there needs to be consideration of how they can be made both affordable and accessible. Consulting with Indigenous custodians and using communities should be central to this process. Furthermore, reparation to Indigenous custodian communities also needs to be addressed.

### Conclusion

It is essential to recognize the significance of thoughtful terminology, the inclusion of Indigenous voices, and the need for reparative justice in the ongoing discourse. These factors are crucial in shaping consent processes that respect the cultural and historical contexts of these substances and build on existing knowledge. Furthermore, as research in this field progresses, novel approaches that embrace interdisciplinary methods, community engagement, diversity, and intersectionality are vital. They should inform standards of practice and oversight including addressing the potential for boundary violations. It is also important to acknowledge that as current treatment approaches are prohibitively expensive they will continue to drive the practice underground, with implications for safety and accessibility. Balancing innovation with ethical considerations will be key to ensuring that the benefits of psychedelic therapies are accessible, equitable, and responsibly integrated into modern medicine.

## Weed and power: the historical canon according to cannabis

The history of psychoactive substances is deeply intertwined with the processes of globalization, influencing both thought and culture across the world.^2^ As scholars like Breen (2019) and Schlag (2021) emphasize, the boundaries between illicit and licit, recreational and medicinal have often been blurred.^2^^,^^63^ Plant-derived psychoactive substances have long been extracted from non-European contexts for trade, curiosity, and medicinal purposes and cannabis is a prime example of this history.

First cultivated over 10 000 years ago by sedentary agrarian societies in Central and Southeast Asia for food, fiber, and medicine, cannabis later spread to Africa, Europe, and beyond, where it became entangled with colonial ambitions.^64^ From the 16th to the 19th centuries, the colonial powers of Portugal and Britain promoted its use to control and commodify global labor forces, including populations in India and those enslaved in Africa and the Americas.^65^^,^^66^

Cannabis was also essential for maritime enterprises, particularly in the British Empire, where hemp was a crucial material for sails and ropes and a significant source of taxable income.^67^ It became linked to illegality via British efforts to preserve state revenue from suppliers and producers attempting to avoid state-imposed fees. In the early 20th century the British Government faced mounting pressure from the US and the League of Nations to curb its sale and production of the plant, leading to it becoming increasingly criminalized.^66^

Beyond its colonial commodification, cannabis has long been regarded for its utility, adopted in spiritual and medicinal practices across cultures throughout history. From Shinto ceremonies around 5000 BCE to Hindu ascetics, Sufi mystics, and Tibeto-Himalayan Tantric Buddhism, its spiritual role is well documented. It has also been linked to Zoroastrian Persian, medieval Greek, and Arab medical practices and remains sacramental for Rastafarian communities. Its widespread proliferation reflects its versatility and global resonance.

Despite its cultural legacy, cannabis regulation has historically been shaped by politics and moral ideologies, often resulting in uneven enforcement, especially in racialized and disadvantaged communities.^66^ Modern regulatory practices have disproportionately impacted African-American, Afro-British, and Indigenous communities despite comparable usage rates with Caucasians.^68^ African-Americans are 4 times more likely to be arrested for cannabis possession, Afro-British in the UK 11.8 times more likely to be sentenced, and similar findings are reflected in other parts of the world.^68^^–^^73^ Although legislative changes in several US states have reduced the number of cannabis-related African-American arrests, the racial gap in these rates has actually grown.^68^ Similarly, drug-related justice reforms unequally impact Indigenous Australians.^74^ This intersection of race, politics, and cannabis regulation illustrates how historical injustices continue to shape who benefits from legal cannabis reforms and who remains at risk of legal repercussions.

These disparities extend beyond the justice system, undermining economic opportunities and community stability. The conflation of migration, ethnicity, criminality, and cannabis use continues to impact public policy and reinforce harmful stereotypes. Sensational media portrayals such as the 1936 film ‘Reefer Madness’, along with various political figures have perpetuated this stigmatization. Notably, US President Nixon’s War on Drugs exploited racial prejudices and used moral hyperbole to capitalize on intergenerational attitudinal divides, discredit the anti-war movement, and ultimately influence global drug policies. Cannabis continues to occupy a complex and contradictory status and remains the most commonly used illicit drug worldwide.

Historical biases inevitably skew scientific research, funding, and public policy. While previous research heavily focused on the negative effects of cannabis, more recent data challenges some of their conclusions. Post-2018 findings in the US and Canada indicate that legalization did not result in increased usage or higher rates of hospital admissions for problem use as anticipated.^75^ In Canada, crime rates remained stable, and cannabis-related youth offenses dropped by 50%.^75^ Furthermore, the causal link between cannabis and schizophrenia is also being re-considered.^75^ As global cannabis legitimization continues to evolve, it is essential to avoid letting current attitudes unduly shape research and policy. Recognizing inherent issues within research funding and scholarship, and the responsibilities of researchers, publishers, and policymakers alike will help refine the evidence-based and inform equitable policies and practices. Moreover, healthcare providers and policymakers must acknowledge that medicinal cannabis cannot be divorced from its broader legal and social history.

### The Green puzzle: unraveling chemical complexity and pharmacological approaches

Cannabis contains over 568 compounds including bioactive phytocannabinoids, non-cannabinoid phenols, terpenes, flavonoids, and alkaloids.^76^ Scientific research has established a good understanding of the endocannabinoid system, the phytocannabinoid delta-9-tetrahydrocannabinol (THC), and to a lesser extent, cannabidiol (CBD). Much remains unknown about the system’s complex interactions with botanical cannabis. First identified in 1988, the endocannabinoid system plays a crucial role in regulating behavioral and physiological processes such as immune response, neuroprotection, cognition, and sleep, making it a promising target for psychiatric treatment.

Yet receptor modulation within this system is intricate. Cannabis metabolites, through a synergistic process known as the entourage effect, alter receptor potencies and contribute to the clinical and subjective effects of cannabis.^77^ The modulatory activities of many cannabinoids, as well as the interactions of terpenes like myrcene, which can generate harmful byproducts when heated, are not fully understood.^78^^,^^79^

Further complicating the science is the distinct pharmacological profile of synthetic cannabinoids. Unlike phytocannabinoids which act as partial agonists, synthetic cannabinoids such as dronabinol and nabilone are full agonists. They have a greater receptor affinity and with their effects unmediated by other bioactives, have a greater psychoactive effect. Understanding the differences between cannabis medicines is critical to preventing oversimplification, misleading comparisons, and inaccurate treatment inferences.

The complexity also extends to the cultivation and chemical diversity of cannabis. Hybridization has resulted in a vast array of chemotypes with significant variation in the chemical composition of each product. Inconsistent regulation of agricultural practices and batch-to-batch consistency further complicates dosing.^79^^–^^81^ For example, regulated dried cannabis in Canada shows THC and CBD concentrations range from 0.01% to 30%.^82^ Other studies have replicated these findings for the major cannabinoids, highlighting the challenges of standardizing botanical cannabis medicines.^83^

Knowledge of pharmacokinetics is also limited, especially regarding different routes of administration. While most controlled studies focus on smoked cannabis containing 6% THC, this does not reflect current market trends where THC percentages are much higher.^79^ In practice, over two-thirds of cannabis is consumed via inhalation, followed by oral ingestion.^84^ Inhaled cannabis is rapidly absorbed, allowing easier dose titration and remains an exceedingly popular mode of delivery despite the established health risks and the loss of THC through combustion.^85^ Furthermore, differences in onset and absorption could significantly affect clinical utility, highlighting the need for more research.

CBD and THC are known to inhibit metabolic enzymes and cannabinoids can interact with a range of common medications.^86^^,^^87^ Notable interactions include those with opioids and antiepileptic medications, where cannabis can exaggerate side effects such as somnolence.^88^ CBD has been shown to increase SSRI concentrations although its clinical significance remains unclear.^89^ Understanding the pharmacokinetic profiles of cannabinoids is essential for evaluating their risks, benefits, and appropriate clinical applications and guiding future research.

Self-titration seems to be the prevalent method for dosing which has the potential to lead to misuse, diminish patient confidence, and expose patients to a greater risk of harm and side effects, especially across different formulations where onset and duration of effect vary. Additionally, dose–response relationships are not always linear. For instance, animal models suggest low-dose THC is anxiolytic, while higher doses can be anxiogenic.^90^ Addressing these issues and optimizing dosing requires a sophisticated approach such as chemoinformatics to create chemical profiles that predict cannabinoid receptor activity.^83^ This, along with stricter regulatory measures may provide the clarity needed to safely and effectively repurpose cannabis for psychiatric treatments.

Navigating cannabis formulations can be overwhelming. Botanical products range from isolates to broad and full-spectrum, classified by species; indica, sativa, or hybrid and proportion of common phytocannabinoids, such as CBD, THC, cannabigerolic acid, and cannabichromene. THC and CBD concentrations are typically listed as percentages or in milligrams per dose for edibles and terpene profiles are often provided too.^79^ However, treatment choices driven by tweaking isolated effects without integrating real-world evidence echo outdated chemical imbalance models, ultimately doing a disservice and fostering disillusionment with medical professionals. While we are only beginning to grasp cannabis’s chemical complexity, this underscores the need for rigorous research to define its medical applications. The interplay of various chemical components directly influences safety, efficacy, and therapeutic potential and without thorough investigation, we risk prescribing in the dark.

### Prescribing in motion: cannabis, psychiatry, and the evidence gap

Despite cannabis medicines securing public attention and prescribing gaining momentum, several discrepancies remain. Poorly tolerated across multiple conditions, with adverse effects commonly reported compared to placebo and active drugs, current evidence supports the use of cannabis medicines for certain indications where other treatments have failed.^91^^–^^94^ Adverse effects, often tied to THC dosage, include worsened cognition and exacerbation of both common and severe psychiatric symptoms in conditions like depression and psychosis.^92^^,^^95^

In the psychiatric context, there appears to be a disconnect between current prescribing practices and the existing evidence. A large body of high-quality data shows it is a risk factor for common psychiatric disorders, while the therapeutic evidence for its use is generally weak and inadequate with multiple systematic reviews concurring that there are safer and better-tolerated options available.^96^^–^^101^ Despite this, cannabis is widely prescribed for psychiatric conditions, frequently self-administered by those seeking relief where other treatments seem inadequate or inaccessible, even if this contradicts professional medical advice. After chronic pain, anxiety is the second most common reason for cannabis prescription in Australia. Since its availability in 2019, around 150 000 prescriptions have been approved for anxiety including 5600 in July 2024 alone.^102^ The broad application of cannabis for these conditions may represent a catch-all approach to various psychiatric symptoms and lack specificity in addressing underlying problems. This could lead to delayed effective treatment, overmedication, and undue harm.

Widespread prescribing raises the possibility of inappropriate use, ambiguous diagnoses, or a large burden of untreated psychiatric illness. The complexities of cannabis as a medicine, its varied chemical composition, the lack of consistent regulation, and the early stage of research, further complicate the situation. There is a distinct lack of evidence-based guidelines for prescribing cannabis in psychiatric contexts, particularly when dealing with psychiatric comorbidities, such as chronic pain, which is often associated with poorer prognosis and has overlapping genetic and epidemiological factors.^103^ While there is hope that future clinical trials will clarify optimal dosing and product types, current research remains insufficient.^104^

Notably, in Australia, young males are the primary users of prescribed cannabis for psychiatric purposes, often opting for flower products. These patterns align more with recreational cannabis use than with the demographic distribution of mental illnesses.^104^ In this new context, there is a need to address the large number that self-medicate with cannabis. Without well-informed, evidence-based guidance, these patients may be navigating treatment options without the necessary support, potentially leading to suboptimal outcomes and further complicating their conditions.

There is also some overlay with psychedelics whereby researchers have begun to recognize the influence of extra-drug factors. Early research indicates that therapeutic outcomes may be influenced by an interplay of therapeutic and subjective effects, further emphasizing the complex dynamics at play in these treatments.^105^^–^^107^ A small study of adults undergoing an acute stress test showed that their prior beliefs about the anxiolytic properties of CBD significantly influenced outcomes.^104^^,^^108^ Researchers have also noted that contextual factors appear to influence not only effectiveness but also drug dependency.^63^^,^^108^^,^^109^ There is some evidence suggesting therapeutic benefit when cannabis is used in conjunction with psychedelic-assisted approaches.^105^^–^^107^ Understanding these factors is crucial for optimizing the therapeutic potential of cannabis and other psychoactive substances in psychiatric care as incautious prescribing will compromise quality of care.

### At the crossroads: care ethics, skepticism, and consent

Best medical practice is grounded in scientific evidence and patient-physician partnership to enable informed decision-making. These same principles apply to cannabis medicines as they do with any other treatment. Doctors may not feel equipped to advise patients due to the gaps and ambiguities in the evidence, concern for causing harm, greater familiarity with other treatments, and shifting cannabis policies. In a recent survey of 640 Australian general practitioners, only 29% reported feeling comfortable discussing cannabis medicines despite 62% having received patient inquiries in the last 3 months.^110^ Indeed, a lack of health professional education has been a significant issue.^111^^,^^112^ It is however the physician’s responsibility to become educated to appropriately advise and inform. Knowledge of the specific evidenced indications and the specific harms as they apply to an individual or subpopulation is essential, in addition to remaining open to new and evolving evidence.^113^ Some have proposed frameworks for cannabis management and education, along with the inclusion of education in the health professional curriculum.^113^^,^^114^ Like with all other treatments, the same standards of care should apply; including history, examination, education, and continued care planning.^95^

Balancing partnership and responsibility is particularly challenging in emerging psychoactive treatments, where clinical research is still evolving. When patient preferences for cannabis-based therapies conflict with a physician’s duty to ensure safety, the lack of comprehensive evidence further complicates decision-making. Physicians must navigate these waters carefully, respecting patient choices while rigorously evaluating risks and benefits based on limited data. The unregulated use of psychoactive substances carries higher risks, including social and physical harms. Medical supervision, promoting standardization and oversight can mitigate this but it must be weighed against the medicalization of recreational use for treatments lacking clear evidence. This underscores the need for ongoing research to fill the gaps in our understanding of cannabis’s therapeutic potential and safety profile. Aside from the potential harms, introducing treatments without strong evidence or clear goals diverts resources from other pressing healthcare priorities.^115^

In the cultural zeitgeist, cannabis and other psychoactive substances are emerging as medical treatments, and lifestyle choices yet attitudes remain polarized. The link between both criminality and wellness affects public policy and personal, and therapeutic interactions. This mirrors broader societal resistance to contentious yet evidence-based approaches to substance use like pill testing and injecting rooms as well as attitudes to those seeking drug treatment programs. On the other hand, the notion that cannabis is natural and therefore inherently safe, the belief that barriers to accessing cannabis treatment for distressing symptoms are unsympathetic and the focus on symptom relief rather than treatment of specific conditions all reflect challenges in the way new psychoactive treatments are perceived and portrayed.^115^ To maintain an effective therapeutic alliance it is essential that personal biases are set aside and that patient well-being is prioritized alongside rigorous scientific evidence.^115^ This balance helps mitigate the risk of draconianism, stigmatization, and trivialization of people’s experiences ensuring that the focus is not solely on favoring gold-standard evidence from RCTs, but also on subjective wellness.

When prescribing treatments like cannabis where the evidence is limited several key practices must be followed to ensure ethical and effective care. Comprehensive education for informing consent, covering both known and potential unknown risks, clearly defining treatment goals and realistic outcome expectations, and implementing robust risk management strategies encompassing regular monitoring schedules and a planned exit strategy are all critical components of a responsible prescribing protocol. Together, these practices help safeguard patient well-being while exploring the potential benefits of emerging treatments like cannabis.

### Chronic choices: prescribing dependence and the opioid parallel

While the endocannabinoid and opioid systems are distinct, both influence the brain’s reward circuits and affect afferent transmission and emotional responses to pain. Their shared capacity to modulate affect and adaptive motivation explains their role in pain relief but also raises concerns about potential dependency. Careful prescribing is necessary, especially given past experiences with opioid overprescription. Opioid treatment for chronic pain was initially supported by limited evidence, which ultimately led to poor treatment outcomes and serious global health crises involving overdose and dependence.^115^

While the risk of cannabis dependence after any lifetime exposure is lower than for alcohol and tobacco; 8.9% compared with 22.7% and 67.5% respectively, cannabis remains the most widely used illicit substance globally with an estimated 4% of the population aged 15–65 years having used it at least once in 2019.^1^^,^^116^ Understanding the dependence potential of cannabis medicines is critical, especially since most existing data pertains to recreational use, which differs in key aspects.^117^ For instance, cannabis medicines are linked to lower severity of other drug use and reduced frequency of alcohol use disorder compared with recreational cannabis use.^63^ However, problem use is still reported in 21.2% of those prescribed cannabis medicines for chronic pain.^117^ High-dose users report greater pain severity and negative affect, mirroring patterns seen with chronic opioid use.^118^ This highlights concerns about adaptive homeostasis, where long-term use shifts baseline symptom responses, moving them in the opposite direction of the drug’s acute effects. This poorly understood mechanism holds significant ethical implications, relevant to all drugs of dependence. Nonetheless, cannabis medicines may still offer a safer alternative for chronic pain management compared to opioids and pregabalin and a manageable level of dependence may be acceptable for some.^63^

To ensure safe prescribing, more needs to be known to guide screening for risk of dependency, ensure adequate monitoring and education, develop tailored treatment, and balance patient needs over potential harms. As much of medical cannabis use is chronic, a longitudinal understanding of the dependency risks is important to establish.^63^

### Regulating the green rush: systemic issues, commercial influence, and quality control

Cannabis is the most widely used regulated drug today with an estimated 300 million users worldwide (3.86%).^119^ Understanding the broader systemic and regulatory issues is essential. Orenstein et al. (2018) suggest adopting lessons learned from tobacco regulation to improve cannabis oversight, particularly in areas like labeling, packaging, and product information.^120^ Plain packaging for instance offers several advantages, enhancing clarity and reducing brand-driven misinformation which often targets vulnerable populations. Prohibiting packaging that appeals to children or makes lifestyle and aspirational pitches would help moderate public perception and curb potential harm in vulnerable populations. Moreover, standardized warnings and clear product information can educate people on risks, including dependence potential.^120^ Without such regulations, individual dependence issues may be exacerbated by a lack of systemic oversight and inadequate consumer information.

While global regulations are rapidly shifting, some of the existing labels, packaging, and product names are intentionally misleading and make clear appeals to recreational use and intoxication. Cannabis medicine names like Superbly Cozey and Zurple Punch promise contradicting tailored experiences, while others evoke culinary tones like Queen Sangria and Key Lime Kush. Still others have distinctly humorous, nostalgic, and child-oriented appeals like Lemon Sherbet, BuBBlelicious, and Girl Scout Cookies. Straying from the potential medical application of certain bioactive components, brand websites and product names of prescription cannabis medicines emphasize the flavor terpenoids imbue and the color flavonoids impart.^77^ Product catalogues allow subscribed users to view and add personal product reviews, reminiscent of current alcohol, and fine food promotion and brand engagement tactics.

The alcohol, tobacco, ultra-high-processed food, and opioid industries have demonstrated that market profit usually conflicts with public health interests.^121^^,^^122^ Market analysts have postulated that heavy investment in cannabis production by transnational alcohol and tobacco companies enables them to strategically expand and capture related markets and technologies such as cannabis drinks and vaping products. This is concerning given that the so-called commercial determinants of health, that is, commercial and political practices, unhealthy commodities, and corporate influence on policy, significantly underpin noncommunicable diseases which are now the leading cause of morbidity and mortality worldwide.^123^ In the broader context of commercial influence, there is a need for rigorous regulatory oversight to protect public health.

Policymakers must adapt to the changing ways in which people seek and receive health information. Online platforms and social media have become primary sources where information is sought and sharing is promoted.^124^ Cannabis businesses have a strong online presence, with advertisements frequently appearing on these platforms.^125^ Instances of advertising standards violations are common and young people are particularly susceptible, highlighting the ineffectiveness of current age restrictions.^126^ Additionally, online media algorithms are designed to sustain attention, which is more attuned to consumerism than disseminating medical information. The enforcement of regulations remains inadequate in this multibillion-dollar industry where strategic corporate policy violations can be profitable.^127^ Therefore, continuous monitoring and thoughtful governance are essential to maintaining an effective regulatory framework.

Commercial interests, shifting public opinion, relaxed policies, and increased physician familiarity may explain the rise in cannabis prescribing in recent years.^128^ However, increased prescribing does not necessarily equate with effective treatment. Studies highlight a disparity between clinical guidance and actual prescribing practices, suggesting that medicinal cannabis may be used as a stop-gap for conditions with no adequate treatments or where existing treatments have burdensome side effects. Furthermore, prescribing trends may be driven by a feedback loop where past practices influence current decisions, often with little regard for treatment efficacy or robustness of evidence.^104^

An important distinction in the medical cannabis market around the world is that prescribing laws and subsequent relaxing of accessibility regulations have been driven by patient demand rather than evidence basis. There is also variability between countries in the degree of medical oversight. While less oversight can improve cost and accessibility, it has implications for diversion and adverse outcomes.^129^ Additionally, inconsistent legislation across states, and even contradictions between state and federal laws in the US, create challenges for policy and regulation.

Policy decisions can also shape public perceptions. This can lead to the misconception that widespread changes in cannabis legislation equate to treatment endorsement, despite the lack of evidence on efficacy. In Australia, prescription cannabis has been rolled out on compassionate grounds, bypassing standard safety, and effectiveness evaluations. Similarly, low-dose CBD products have been approved for over-the-counter sale in Australia and Britain based solely on safety, potentially misleading consumers to assume these products are effective.^130^

Media portrayal further influences public opinion and can ultimately drive policy changes. This has been evident in the sphere of medical cannabis as well as broader cannabis legislation. While articles focusing on gatekeeping and patient rights appeared to subside with policy changes, Gunning and Iles (2021) highlight the need for a more nuanced, evidence-based media approach that not only educates the public on the appropriate use of cannabis medicines but also represents the interests of marginalized groups, including those with disabilities.^131^

Regulatory oversight in the cannabis industry is essential for ensuring the safety, quality, and efficacy of medical cannabis products. Precision in dosage, purity, and accurate labeling directly impact patient outcomes, making quality assurance a critical component of cannabis medicine. However, significant issues have been identified in these areas, including the presence of contaminants like heavy metals, carcinogens, and solvents such as butane in cannabis concentrates.^82^ Microbial contamination is also a concern, particularly for immunocompromised patients, leading to widespread pesticide use, which poses additional risks for those with neurological conditions.

A study in the US highlighted alarming inaccuracies in the labeling of 75 randomly selected medicinal cannabis edibles. THC content was underreported in 23% of products, overreported in 60%^81^ and none accurately reflected CBD content. These inaccuracies result in treatment ineffectiveness, adverse events, and inconsistent dosing, turning titration into a batch-to-batch guessing game. Similar issues have been reported in the UK for over-the-counter cannabis products.^132^

Quality control failures have led to product recalls in multiple countries, including Canada, Australia, the US, and the Netherlands. Some gray market brands capitalizing on the growing demand for cannabis and other plant-derived psychoactive substances as lifestyle and wellness products further complicates the regulatory landscape. Notably, two brands faced recall after their products were found to contain unlisted DMT analogues and unexpectedly high levels of THC. Substances that were not legally permitted in those jurisdictions.^133^^,^^134^ These incidents underscore the ethical imperative for stringent regulatory oversight in the industry. Proactive regulation in Uruguay caps THC concentrations, aiming to protect its consumers. Without such measures, there is a significant risk to consumer safety, undermining the therapeutic potential of cannabis and compromising the trust that patients place in medical treatments. Ensuring that cannabis products are safe, effective, and accurately labeled is not just a matter of regulation but a critical aspect of ethical medical practice.

### Many medicines, one plant: harnessing research and clinical potentials

Cannabis medical research is fraught with many of the same complexities affecting psychedelic research. Study design heterogeneity, such as primary outcome and disease definition, is confusing when trying to draw definitive conclusions about the efficacy and safety of cannabis-based treatments. This heterogeneity is further compounded by differences in study populations, dosages, forms of cannabis used, and the specific cannabinoids being investigated, making it difficult to standardize findings across different definitions and applications. This has flow-on effects for research synthesis where combining multiple small studies to improve power often gives rise to misleading results. Not ironing out these issues hinders the ability of future research to inform decision-making for physicians and patients alike.

Comparing cannabis use across different contexts also presents research challenges. Studies in countries with strict regulation and quality control may differ greatly from those in countries with less oversight. The impact of legal, regulated environments versus illicit, unregulated markets also influences use patterns, efficacy, and risks, leading to varied outcomes. Historical research that viewed cannabis use as pathological and criminal will have affected hypotheses, definitions, parameters, and research attitudes and approaches. Additionally, in countries with legitimized recreational cannabis use, the profile of individuals seeking prescription cannabis may differ, complicating the generalizability of findings. These contextual variations raise important questions about the applicability of research findings across diverse legal and regulatory frameworks and historical and contemporary contexts.

Cannabis is not one treatment but many. It contains a multitude of bioactive ingredients. Specific strains, composition, preparation, and guidelines all contribute to the therapeutic effect. The entourage effect further complicates research efforts. Isolating the effects of individual components versus the whole plant poses significant challenges to be considered in both research development and clinical practice. Moreover, establishing comparative efficacy is essential. The high costs and logistical complexities of researching plant-based medicines, which cannot easily be patented, dissuade pharmaceutical companies from investing, as any advancements benefit competitors. This creates a tragedy of the commons scenario where a lack of investment can stifle progress.

Given these challenges, there is a pressing need for novel research approaches. Open-label extension studies, which allow participants of an RCT to enroll in an unblinded extension where all subjects are given the active drug could provide valuable safety and tolerability data.^79^ Harnessing registries, observational studies, digital monitoring, and infodemiology could also help gather comprehensive data on the effects of cannabis in various populations and inform potentially fruitful avenues for clinical trials as well as supporting policy and decision-making.^77^^,^^135^^,^^136^ Involving patients and users in the research process through co-production and co-design can ensure that studies align with the needs and experiences of those using cannabis with therapeutic intent and help determine endpoints and outcome measures.^79^^,^^137^ Furthermore, utilizing users in the dissemination of information can enhance the reach and relevance of research findings, making them more accessible to the broader public and providing greater assurance in terms of relevance to real-world experiences.^92^

While it has not entered circulation like other pharmaceuticals of its era, the same rigorous standards should apply to cannabis medicine research. This includes the areas of drug development and regulatory science, safety versus efficacy studies, and public health evaluation. Population health analysis will need to address abuse liability, workplace, and driving safety as well as cost and logistical factors. As estrogen directly modulates the endocannabinoid system, special attention should be given to understanding these implications including for endometriosis, hormone-dependent cancers, and broader impacts on safety, efficacy, and dependence.^138^ Additionally, the role of cannabis in psychiatry remains problematic, warranting further research to clarify its potential benefits and risks.

### Conclusion

Cannabis is deeply entangled with its historical context, rooted in globalization and politics which have contributed to persistent health and justice disparities. It continues to occupy a paradoxical space reflected in attitudes, policies, and health implications. As a plant with numerous bioactive compounds that act synergistically, cannabis medicines are not one treatment but many, a complexity that must guide both research and therapeutic strategies. Regulation around prescribing, quality control, and marketing is crucial to safeguard the public and address the challenges posed by its growing popularity. The current explosion in prescribing and public interest must be balanced with lessons learned from the opioid crisis and other regulated substances of dependence, all while considering the potential harms and benefits. Patient autonomy should be respected within a framework that prioritizes safety and avoids the medicalization of dependence. Finally, adaptive and innovative approaches are needed to address the gaps in current research and ensure cannabis is integrated responsibly into medical practice.

## Stimulating debate: tracing the legacy and ethics of stimulant use

The Chinese ephedra plant has been used in Chinese traditional medicine for over 5000 years, primarily to treat bronchoconstriction, and upper airway congestion with amphetamine first isolated from the plant in 1885. It was subsequently independently synthesized and sold as a pharmaceutical decongestant and by the 1930s researchers had come to recognize its stimulant properties.

In World War II coalition forces on both sides experimented with its use for wakefulness and performance-enhancing and it later became a standard issue for both British and German forces. It has been argued that in the military context, these stimulants inspired strength, endurance, and vigilance. The consumption of the methamphetamine product, Pervitin has been linked with the aggressive German military combat operations up until 1940.^139^ It is estimated that post-war, some 16 million US soldiers had been exposed to amphetamine tablets.^140^

Amphetamines were subsequently rebranded for civilians to treat depression and weight loss, the seductive ‘rainbow diet pills’, were heavily marketed to prescribers and the public alike with inflated benefits, containing numerous active excipients to counter its various side effects.^141^ By the 1950s and 60s, due to widespread concerns about dependence and adverse health effects, amphetamines became increasingly restricted. They came to be associated with crime syndicates, notably the Yakuza in Japan, as well as subcultures like the sharp-dressing Mods, known for their aspirations of creative and financial freedom. By the 1990s stimulants had become heavily regulated in most countries around the world. Stimulants have had continued currency for their alerting and performance-enhancing properties, notoriously used by elite athletes, shift workers, party-goers, and college students alike.

### Stimulants for psychiatric treatment: overprescribing and diagnostic inflation

In psychiatric practice, stimulants are primarily used to treat disorders of attention and wakefulness, overlapping with the broader group of both traditional and recreationally used stimulants including caffeine, nicotine, mate, coca, khat, kratom as well as illicit derivatives such as methamphetamine and cocaine.

Stimulant medications have also been proposed as potential agents to meet some of the gaps where current psychiatric treatments fall short, for example in treatment-resistant depression, negative symptoms of schizophrenia, cognitive disorders such as dementia, as well as combating side effects of other psychotropics such as weight gain, sedation, and sexual dysfunction.

The risk of repurposing stimulants to fill unmet needs in psychiatry is twofold. Firstly, off-label prescribing with a lack of robust evidence, could be hazardous to patients who may not understand the experimental nature of the treatment. The risks may outweigh the benefits and the long-term consequences are not well understood, for example in relation to schizophrenia and cognitive degeneration. Secondly, there is a high risk of dependency. In for example treatment-resistant depression, the promise of stimulants improving apathy and providing symptom relief during the onset latency period for traditional antidepressants, needs to be carefully weighed against the high potential for dependency. This risk is compounded in vulnerable populations with comorbid psychiatric diagnoses, where up to 1 in 4 people with a serious mental illness have a comorbid substance use disorder.^142^^–^^144^

There have long been concerns about stimulant prescribing for normal variations of human behavior and performance. One of the challenges here has been the overlay of social and cultural determinants of diseases, which is strongly the case in ADHD. It has been shown that there are cultural differences in parental sensitivities and social tolerance to externalizing behaviors.^145^^,^^146^ Additionally, cultural bias within the existing diagnostic criteria has been cited as a contributing factor to geographic differences in ADHD diagnosis, further complicating the issue.^146^

The risk with medicalizing behavior and performance is that non-pathological phenomena become labeled as diseases. This approach assumes a clear line between health and disorder, which is rarely the case in neuropsychiatry. There is also no cut point or bimodal distribution of attention, leading to arbitrariness in definition and hence the use of stimulants. Without specific biomarkers and relying on classifying disorders based on a constellation of symptoms, defining disease often depends on interpreting and identifying significant dysfunction and distress, which can be non-objective.^147^

The rise of neurodivergence as an explanation for attention difficulties and underachievement reflects a broader trend where certain diagnoses gain and lose popularity over time, becoming widely accepted explanations for common challenges until another takes their place. While neurodivergence offers a valuable framework, it can also lead to overgeneralizations, diagnostic overshadowing, and the misinterpretation of normal life struggles as clinical disorders. This may result in the inappropriate use of medications as a quick fix in place of therapy or lifestyle changes, particularly when these conditions are heavily publicized or culturally endorsed.

An additional concern is the medicalization of social issues leading to overprescribing. A number of contemporary factors affect diagnosis in this arena. In the case of childhood ADHD, social pressures to behavioral conformity, school governance, and media marketing are all contributors.^147^ Local school funding is increasingly incentivized or influenced by student academic performance. Examples here include the 2001 No Child Left Behind Act in the US and the National Assessment Program – Literacy and Numeracy in Australia. Allocation of additional school or university support is often financed or resourced depending on a diagnosis. In time-constrained, resource-limited nuclear families, and underfunded educational systems, where academic pressures are intense, there is often a shrinking tolerance for behaviors that do not align with expectations, especially in the context of an attention economy. Prescribers may face pressure to make quick decisions and weigh in on resource allocation, often with limited information and there is the risk that medication might be prescribed to address parental distress rather than a child’s actual needs. Like with other neurodevelopmental disorders, ADHD has been proposed as a disorder spectrum or continuum, also conferring a risk of diagnostic overreach.

The use of stimulants for ADHD is also unique in another domain. In no other branch of psychiatry does misdiagnosis and prescription of inappropriate medications become self-reinforcing. However, stimulants are largely pleasurable for most users and enhance mood, attention, focus, and energy. Consequently, many people prescribed these agents will perceive some subjective benefit regardless of whether they do or do not have ADHD and uniquely prescription tends to reinforce the diagnosis whether correct or not.

Neuroenhancement, the use of treatment to improve cognitive, emotional, or behavioral functioning beyond what is considered the realms of disease states, is hotly debated and highlights the issue of treatment boundaries. Sakakibara et al. (2020) argue that it is essential to recognize the ambiguities in treatment, where the coexistence of multiple meanings blurs the boundaries between therapeutic intervention and enhancement.^148^ This is particularly relevant in psychiatry, where psychotropic medications not only manage symptoms of mental illness but also alter human experiences, emotions, and perceptions. For instance, subjective displeasure due to emotional blunting and anxiety was a key factor for antidepressant discontinuation in a third of 896 surveyed study participants.^149^ Sakakibara et al. (2020) go on to argue that neuromodifiers lie on a continuum between treatment and enhancement and overlap with the elements of prevention, pain relief, and pleasure-seeking. This raises ethical questions about the appropriate limits of medical intervention and the potential societal implications of enhancing human capacities beyond their original state. It challenges the distinction between healing and enhancement and prompts a reexamination of what it means to treat versus augment.

Perhaps some of the difficulty in defining treatment in psychiatry arises from its inherent tension, as the field often seeks to reduce the rich complexities of embodied human experience to mental processes. This reductionist approach risks overshadowing the nuanced interplay between mind and body, potentially missing key aspects of human suffering. It raises the question of whether psychiatric clinicians are tasked solely with the medicine of the mind, or if they should also serve as advocates for ethical and humane treatment of the broader human experience, including physical and existential dimensions of suffering.

The argument by Sakakibara et al. (2020) on the continuum of treatment with pleasure-seeking is particularly intriguing, raising the distinction between hedonia and eudaimonia, or immediate pleasure versus self-actualization and fulfillment regardless of circumstances. Psychotherapy is another domain that crosses into these territories in addressing both ease and disease.

One salient point raised by many is the lack of evidence to support that prescribed stimulants such as amphetamine and methylphenidate can fulfill a promise of neuroenhancement.^150^^–^^153^ The findings of the metanalysis of dementia medications in healthy individuals are even more disappointing, showing no improvement in attention, vigilance, concentration, or memory.^154^^,^^155^ Some have argued that optimal function in one domain may mean a trade-off in another.^153^ A meta-analysis of 148 studies found methylphenidate improved academic productivity but not accuracy, while others found improved attention and vigilance may be at the cost of long-term memory, executive functions, and creative thinking.^156^^,^^157^ Others pose that the presumption that stimulants improve cognition is misguided and that more research should focus on the volitional advantage they provide. They are known to treat apathy, improve task enjoyment, and make users more inclined to engage in high-effort, high-reward tasks.^151^ These debates aside, disease treatment should lead the way in terms of resource allocation for medical research and community health initiatives, ensuring that the focus remains on addressing genuine needs over enhancement potentials.

### Recreational use, dependence, and diversion; the psychiatric implications

According to the 2024 United Nations World Drug Report, an estimated 30 million people worldwide use illicit amphetamine-type stimulants, while 23 million use cocaine. Polydrug use among stimulant users is common, compounding the risks involved.^158^ Key concerns include cardiovascular and cerebrovascular complications, blood-borne viruses among injecting users, along with increased utilization of first-line workers, and higher rates of violence and psychosis.^159^ Notably, for those using amphetamines, the risk of developing psychosis is 2.5% while the subsequent risk of conversion to schizophrenia is around one-third.^160^ Given the global prevalence of illicit stimulant use and the strain this places on health care systems, there is a clear ethical duty to ensure that treatment approaches do not worsen these outcomes.

One of the major challenges in addressing illicit stimulant use is the lack of robust, easily implemented treatment options. The current evidence supports contingency and psychosocial management, with a lack of effective pharmacotherapies available.^161^ Although prescribing stimulants as treatment appears to reduce cravings, it does not reduce the frequency of illicit use. The results from antidepressant trials such as mirtazapine to treat dependence have been mixed.^162^ Research in this area has been marred by poor adherence and retention rates.^163^ Additionally, stigma, discrimination, and concerns about lack of confidentiality remain barriers to help-seeking, with women unequally affected.^158^^,^^164^ A perceived lack of need to reduce use also remains an obstacle.^158^ This situation underscores the need for ongoing research and development of more effective treatment strategies that responsibly mitigate these risks.

Treating comorbid ADHD and stimulant use disorder in adults poses added challenges. Non-stimulant medications can prevent diversion, but they do not address substance dependency and untreated ADHD increases the risk of both developing a substance use disorder and stimulant use relapse.^159^^,^^165^

Shifting to a related area, non-medical prescription stimulant use is thought to be most common among those working in high-pressure, cognitively demanding environments, though prevalence within these cohorts has not been established.^166^ A larger body of research has been conducted among USA and European university students, with one systematic review of treatment diversion placing the past month’s prevalence of non-medical stimulant use between 4.15%, and 22.7%.^167^ While perhaps differing in frequency of use, this is more common than the prevalence of the condition for which these treatments were intended. In university students, the desire to enhance academic performance and productivity are chief motivators (50–89%).^168^^,^^169^ While indicative of individual behaviors also reflects broader societal pressures.

A recent systematic review found that 4% to 35% of university students admitted to using prescription stimulants from their own supply for non-medical purposes.^168^ Personal surplus was associated with diversion while most stimulant acquisition occurred within social networks.^170^ Although prescription misuse disorder is uncommon in this cohort the risks from unsupervised use and rates of concurrent substance use remain high.^168^^,^^171^ The issue extends beyond university campuses. A survey of 180 parents revealed that 16% diverted their child’s stimulants, primarily for personal use. The risk was even higher at 33% among parents with suspected or confirmed ADHD.^172^ This highlights the responsibility of prescribers to closely monitor stimulant prescriptions to prevent inadvertently contributing to misuse and associated harms.

Illegitimate online pharmacies offering falsified or illegally procured restricted medicines are also a growing concern.^173^^,^^174^ Outcomes relating to contaminated opioid products have been fatal. A recent study identified 61 of an identified 62 internet pharmacies selling stimulants were illegitimate, all offering unrestricted quantities without prescriptions and in a Good Manufacturing Practice-free environment, what is on the label does not necessarily reflect what is in the pill.^173^ This underscores the need for robust patient education and stricter access regulations to prevent harm.

A related phenomenon is feigning ADHD symptoms to gain access to medications or academic advantages, such as special exam conditions or in some countries, financial benefits like altered student loan fees.^169^ In fact, 20% of adults in one study admitted to fraudulently obtaining stimulants from a doctor.^168^ ADHD diagnosis relies on self-report and collateral information, which can be unreliable and easily manipulated. Psychometric tests for ADHD have been found susceptible to being falsified, whereas personality tests are effective at detecting ADHD malingering and performance validity testing also forms part of the armament.^169^ Many prescribers adopt strategies like the use of long-acting agents and non-stimulant medications when feigning is suspected. However, another study found that at least a quarter of surveyed doctors did not feel adequately equipped to educate patients on the risks of non-medical use and diversion.^168^ To address these issues, increasing public awareness about the dangers of non-medical stimulant use, and routinely adopting ADHD assessment tools that detect malingering, judicious prescribing, and supply monitoring are crucial steps.

### Autonomy, identity, authenticity, and informed consent

Prescribing stimulants to young people with an evolving capacity is complex. Beyond the pressures to conform to parental expectations and societal norms, medication can deeply influence one’s self-concept. As psychotropic medications not only treat symptoms but also affect human sensibilities, stimulants not only shape behavior but also influence thinking and emotions. For young people, this is at a critical time of individuation and identity formation. The narratives surrounding treatment for externalizing behaviors may communicate sentiments of inadequacy and inherent badness, that may become internalized, with profound implications for self-concept and emotional development. As such, it is important to consider how psychotropic medications impact not just behavior, but the broader aspects of an individual’s emerging identity.

While medications are not the first step in treating childhood and adolescent ADHD, they offer a quick response, are easily implemented, and involve fewer moving parts compared with embedding psychosocial strategies in the complex systems surrounding a young person. There is also the matter of available and accessible resources, including skilled clinicians and the resourcing of parents.

Supported by the literature, adolescence, and childhood represent critical periods of brain maturation, including changes in cortical thickness, myelination, and the refinement of neural networks, all of which are crucial for cognitive and emotional development. Stimulants, when introduced during these sensitive periods, have the potential to induce lasting neurobiological changes that can alter neural circuitry and neurotransmitter systems, with a lasting impact on cognitive abilities, emotional regulation, and behavior in adulthood. The potential for stimulants to effect such enduring changes underscores the importance of carefully considering the long-term implications of their use during these formative years.

Recent metanalysis found ADHD medications to be protective for a broad range of functional outcomes.^175^ However, their long-term effects on neurodevelopment remain unclear. Outcome measures are largely inferred from animal models and raise specific concerns about the developmental alterations including changes in neuroarchitecture, dopamine signaling, and dopamine receptor densities.^176^^,^^177^ A phenomenon known as neural imprinting, whereby early drug exposure causes latent effects that manifest after the nervous system matures, has been extensively documented in preclinical animal models, but whether these effects are deleterious remains unknown.^178^^,^^179^ The small number of human studies on methylphenidate effects on neurodevelopment have shown attenuated white matter volume changes and increased functioning in several domains when compared with untreated subjects. However, these studies frequently exclude poor responders, an estimated 30% of which may be attributable to interindividual variability.^178^ Additionally, the majority of studies exclude women and girls, leaving significant gaps in understanding the long-term effects on these populations.

There is a tension between the need for swift action and the desire for quick fixes in ADHD treatment, but the grounding for these decisions is already shaky. A network meta-analysis of 190 studies of ADHD treatment in children and adolescents with a mean age of 10 (range 3–16), found the average treatment duration in studies was only 12 weeks, and sample size and heterogeneity limited the external validity of findings.^180^ While stimulants show short-term benefits.^181^, the prospect of long-term studies reducing prescribing, disincentivizes pharmaceutical investment.^181^ Although a multimodal approach is beneficial across all age groups, the optimism around stimulants and treatment adherence should be approached with caution.^180^^–^^182^

Common concerns for young people prescribed stimulants are their role in choice and decision-making, aspects of self-identity, and control.^183^^–^^186^ Concerns about control related to attention and behavior and emotions, while self-identity themes included acceptance and belonging.^183^ Both these concerns clusters are interrelated. From an attachment theory perspective, a child’s sense of security and self-worth is developed through positive relationships with caregivers. A child with ADHD may perceive their behavior as not acceptable, while outside efforts are made to control or modify them to meet external expectations, leading to challenges in forming a stable sense concept. Furthermore, Ringer et al. (2020) in their qualitative analysis of childhood experiences of ADHD, highlight that according to Eriksonian psychosocial stages, the task of adolescence is to develop a stable and coherent sense of self and personal identity. Other qualitative analyses have highlighted changes in experiences of control as the young person transitions to adulthood.^184^^,^^185^ Combined analyses found that with evolving dominion, firstly decision-making develops in relation to medications, then autonomy, self-management with an emerging internal locus of control.^185^ While these themes may be unsurprising for this life stage, perhaps ADHD and its associated treatments act as an interlocutor in navigating this formative period.

Still, from the perspective of young people, medications do not reliably improve a sense of control.^186^ Relatedly, other studies demonstrate significant short-term symptom improvement with placebo treatment and interestingly, one longitudinal study found that except for social and emotional outcomes, ADHD symptoms improved in adolescents over 6 years, regardless of stimulant treatment.^187^^,^^188^ Some researchers have suggested that ADHD may reflect a delay in behavioral maturation.^189^ These findings suggest that the current understanding of ADHD is likely too simplistic, prompting calls for more nuanced approaches and longitudinal data on this multifaceted condition.

The idea that stimulants prescribed in childhood might undermine moral responsibility for one’s actions has also been debated.^190^ This framework of virtue ethics emphasizes character development and self-control and ties into broader cultural norms that prioritize self-regulation and rationality as markers of moral character. This perspective, however, risks reducing the issue to a matter of willpower and overlooks the complex neurobiological factors that influence behavior. It also implies that a lack of control reflects a deficiency in moral character, a view that is not supported by the qualitative literature. Studies have shown that ADHD medications often negatively impact young people’s self-esteem.^186^ Furthermore, children with ADHD frequently experience a sense of self-ambivalence and lack of belonging, define themselves by others’ perceptions, and relate to an ADHD-defined self.^183^

This connects to the concept of internalized ableism from disability theories, where individuals adopt society’s negative views about their capabilities as intrinsically flawed, not meeting normative standards.^186^ To counter this, a care ethics model is proposed, where individuals are viewed as interconnected and embodied beings with inherent vulnerabilities, moving beyond rigid moral expectations toward a more empathetic understanding of their experiences.

There is an ethical obligation that clinicians ensure they seek consent from the young person, to recognize and respect their emerging capacity to participate in decision-making. This is especially pertinent regarding treatments that impact behavior, emotions, and self-identity. This is grounded in the principle of respect for dignity and autonomy. Conversations should not only focus on the risks, benefits, and realistic expectations of medications but also invite a conversation about experiences and beliefs about medications as well as the impacts on their sense of personhood.

### Conclusions

There is a complex landscape to be navigated when considering the benefits and risks of prescribing stimulants. The challenge of addressing unmet psychiatric needs with limited evidence impacts fully informed consent. The cultural and context-dependent nature of performance and behavior risks medicalizing and medicating potentially normal variations. Additionally, the lines are blurred between what constitutes disease and what distinguishes treatment from enhancement, especially in contexts driven by social and vocational pressures. Its entity, further complicates non-medical use, with a lack of effective pharmacotherapeutic treatments and an ill-defined cohort that uses it for subjective but not always euphoric effects. In the case of prescribing stimulants to children, matters are very complex, where questions remain about the impacts on the developing brain and a child’s emerging identity are not fully understood. A young person’s evolving capacity to consent and the dynamics of the child-carer relationship further complicate these decisions. A careful, ethically informed approach is required that prioritizes the long-term well-being of the young person. In conclusion, the medical use of stimulants requires a nuanced and cautious approach, balancing the potential benefits with the significant ethical challenges posed by their use in both medical and non-medical contexts.

## Future directions and global ethical considerations

While manipulating brain chemistry offers valuable therapeutic potential, it cannot fully address the complexities of the human mind. Accordingly, searching for pharmacological solutions to the profound and existential challenges people face could erode psychiatric care.

Rigorous ethical codes are crucial, but cannot take the place of an active, thoughtful, creative approach to the moral responsibilities clinicians face. These extend beyond simply prescribing. Medically adapted psychoactive substances harbor a potential for mistreatment and misuse affecting not only individuals but also communities. Meanwhile, they also offer the potential for paradigm-shifting changes that could radically progress the field of psychiatry. In holding the potential to cause great harm, they call for an ethically intelligent approach. This means an active process of continuous awareness, ongoing questioning, and personal responsibility alongside international cooperation in ethical oversight.

This ethical responsibility will need to straddle both non-medical and medical contexts, acknowledging that some non-medical use is driven by perceived benefit and spiritual intent and is not solely recreational. It perhaps calls us to face these sophisticated problems with sophisticated solutions and accessibility will be a key domain of focus. Relatedly, precision psychiatry should not just focus on individual bodies but also embrace system complexity, acknowledging that people are interconnected and that solutions should be holistic, rather than individualistic.

## References

[1] WHO. *World health statistics 2023: monitoring health for the SDGs, Sustainable Development Goals.* 2023:119. 19 May 2023. https://www.who.int/publications/i/item/9789240074323

[2] Breen B. The age of intoxication: origins of the global drug trade. University of Pennsylvania Press; 2019.

[3] Franklin NR, Giorgi M, Habgood PJ, et al. Gilparrka Almira, a rock art site in Mithaka Country, southwest Queensland: cultural connections, dreaming tracks and trade routes. Archaeol Ocean. 2021;56(3):284–303.

[4] Schlag AK, Aday J, Salam I, Neill JC, Nutt DJ. Adverse effects of psychedelics: from anecdotes and misinformation to systematic science. J Psychopharmacol. 2022;36(3):258–272. 35107059 10.1177/02698811211069100 PMC8905125

[5] Blatchford E, Bright S, Engel L. Tripping over the other: could psychedelics increase empathy? J Psychedelic Stud. 2021;4(3):163–170.

[6] Gattuso JJ, Perkins D, Ruffell S, et al. Default mode network modulation by psychedelics: a systematic review. Int J Neuropsychopharmacol. 2023;26(3):155–188. 36272145 10.1093/ijnp/pyac074 PMC10032309

[7] Ko K, Knight G, Rucker JJ, Cleare AJ. Psychedelics, mystical experience, and therapeutic efficacy: a systematic review. Front Psychiatry. 2022;13:1–12 10.3389/fpsyt.2022.917199 PMC9340494 35923458

[8] Griffiths RR, Richards WA, McCann U, Jesse R. Psilocybin can occasion mystical-type experiences having substantial and sustained personal meaning and spiritual significance. Psychopharmacology. 2006;187:268–283. 16826400 10.1007/s00213-006-0457-5

[9] Ross S, Bossis A, Guss J, et al. Rapid and sustained symptom reduction following psilocybin treatment for anxiety and depression in patients with life-threatening cancer: a randomized controlled trial. J Psychopharmacol. 2016;30(12):1165–1180. 27909164 10.1177/0269881116675512 PMC5367551

[10] Roseman L, Nutt DJ, Carhart-Harris RL. Quality of acute psychedelic experience predicts therapeutic efficacy of psilocybin for treatment-resistant depression. Front Pharmacol. 2018;8:974. 29387009 10.3389/fphar.2017.00974 PMC5776504

[11] Luckenbaugh DA, Niciu MJ, Ionescu DF, et al. Do the dissociative side effects of ketamine mediate its antidepressant effects? J Affect Disord. 2014;159:56–61. 24679390 10.1016/j.jad.2014.02.017 PMC4065787

[12] Doblin R. Pahnke’s “Good Friday experiment”: a long-term follow-up and methodological critique. J Transpersonal Psychol. 1991;23(1):1–28.

[13] De Vos CM, Mason NL, Kuypers KP. Psychedelics and neuroplasticity: a systematic review unraveling the biological underpinnings of psychedelics. Front Psychiatry. 2021;12:1–17. 724606. 10.3389/fpsyt.2021.724606 PMC8461007 34566723

[14] Gumpper RH, Roth BL. SnapShot: psychedelics and serotonin receptor signaling. Cell. 2023;186(1):232–232. 36608655 10.1016/j.cell.2022.12.020

[15] Ly C, Greb AC, Cameron LP, et al. Psychedelics promote structural and functional neural plasticity. Cell Rep. 2018;23(11):3170–3182. 29898390 10.1016/j.celrep.2018.05.022 PMC6082376

[16] Nichols CD. Psychedelics as potent anti-inflammatory therapeutics. Neuropharmacology. 2022;219:109232. 10.1016/j.neuropharm.2022.109232 36007854

[17] Husain MI, Ledwos N, Fellows E, et al. Serotonergic psychedelics for depression: what do we know about neurobiological mechanisms of action? Front Psychiatry. 2023;13:1076459. 10.3389/fpsyt.2022.1076459 PMC9950579 36844032

[18] Cooper T, Seigler MD, Stahl S. Rapid onset brain plasticity at novel pharmacologic targets hypothetically drives innovations for rapid onset antidepressant actions. J Psychopharmacol. 2023;37(3):242–247. 36988262 10.1177/02698811231158891 PMC10076337

[19] Kwan AC, Olson DE, Preller KH, Roth BL. The neural basis of psychedelic action. Nat Neurosci. 2022;25(11):1407–1419. 36280799 10.1038/s41593-022-01177-4 PMC9641582

[20] Rothman RB, Baumann MH. Therapeutic and adverse actions of serotonin transporter substrates. Pharmacol Ther. 2002;95(1):73–88. 12163129 10.1016/s0163-7258(02)00234-6

[21] Fortier JH, Pizzarotti B, Shaw RE, Levy RJ, Ferrari G, Grau J. Drug-associated valvular heart diseases and serotonin-related pathways: a meta-analysis. Heart. 2019;105(15):1140–1148. 31129607 10.1136/heartjnl-2018-314403 PMC7043398

[22] Droogmans S, Cosyns B, D’haenen H, et al. Possible association between 3, 4-methylenedioxymethamphetamine abuse and valvular heart disease. Am J Cardiol. 2007;100(9):1442–1445. 17950805 10.1016/j.amjcard.2007.06.045

[23] He D-Y, McGough NN, Ravindranathan A, et al. Glial cell line-derived neurotrophic factor mediates the desirable actions of the anti-addiction drug ibogaine against alcohol consumption. J Neurosci. 2005;25(3):619–628. 15659598 10.1523/JNEUROSCI.3959-04.2005 PMC1193648

[24] Marton S, González B, Rodríguez-Bottero S, et al. Ibogaine administration modifies GDNF and BDNF expression in brain regions involved in mesocorticolimbic and nigral dopaminergic circuits. Front Pharmacol. 2019;10:193. 30890941 10.3389/fphar.2019.00193 PMC6411846

[25] Noller GE, Frampton CM, Yazar-Klosinski B. Ibogaine treatment outcomes for opioid dependence from a twelve-month follow-up observational study. The Am J Drug Alcohol Abuse. 2018;44(1):37–46. 28402682 10.1080/00952990.2017.1310218

[26] Mash DC, Duque L, Page B. Ibogaine detoxification transitions opioid and cocaine abusers between dependence and abstinence: clinical observations and treatment outcomes. Front Pharmacol. 2018;9:345105. 10.3389/fphar.2018.00529 PMC5996271 29922156

[27] Koenig X, Hilber K. The anti-addiction drug ibogaine and the heart: a delicate relation. Molecules. 2015;20(2):2208–2228. 25642835 10.3390/molecules20022208 PMC4382526

[28] Alper K, Bai R, Liu N, et al. hERG blockade by iboga alkaloids. Cardiovasc Toxicol. 2016;16:14–22. 25636206 10.1007/s12012-015-9311-5

[29] Cameron LP, Olson DE. The evolution of the psychedelic revolution. Neuropsychopharmacology. 2022;47(1):413. 34400786 10.1038/s41386-021-01150-y PMC8616895

[30] Zhang J, Yao W, Hashimoto K. Arketamine, a new rapid-acting antidepressant: a historical review and future directions. Neuropharmacology. 2022;218:109219. 10.1016/j.neuropharm.2022.109219 35977629

[31] Vargas MV, Meyer R, Avanes AA, Rus M, Olson DE. Psychedelics and other psychoplastogens for treating mental illness. Front Psychiatry. 2021;12:727117. 34671279 10.3389/fpsyt.2021.727117 PMC8520991

[32] Peters J, Olson DE. Engineering safer psychedelics for treating addiction. Neurosci Insights. 2021;16:26331055211033847. 10.1177/26331055211033847 PMC8295933 34350400

[33] Feduccia A, Agin-Liebes G, Price CM, Grinsell N, Paradise S, Rabin DM. The need for establishing best practices and gold standards in psychedelic medicine. J Affect Disord. 2023;332:47–54. 37003433 10.1016/j.jad.2023.03.083

[34] Luoma JB, Chwyl C, Bathje GJ, Davis AK, Lancelotta R. A meta-analysis of placebo-controlled trials of psychedelic-assisted therapy. J Psychoactive Drugs. 2020;52(4):289–299. 32529966 10.1080/02791072.2020.1769878 PMC7736164

[35] Thrul J, Garcia-Romeu A. Whitewashing psychedelics: racial equity in the emerging field of psychedelic-assisted mental health research and treatment. Drugs: Educ Prev Policy. 2021;28(3):211–214.

[36] Paradies Y. A systematic review of empirical research on self-reported racism and health. Int J Epidemiol. 2006;35(4):888–901. 16585055 10.1093/ije/dyl056

[37] Hovmand OR, Poulsen ED, Arnfred S, Storebø OJ. Risk of bias in randomized clinical trials on psychedelic medicine: a systematic review. J Psychopharmacol. 2023;37(7):649–659. 37403379 10.1177/02698811231180276 PMC10350724

[38] Bouso JC, González D, Fondevila S, et al. Personality, psychopathology, life attitudes and neuropsychological performance among ritual users of ayahuasca: a longitudinal study. 2012; 10.1371/journal.pone.0042421 PMC3414465 22905130

[39] Aday JS, Davis AK, Mitzkovitz CM, Bloesch EK, Davoli CC. Predicting reactions to psychedelic drugs: a systematic review of states and traits related to acute drug effects. ACS Pharmacol Transl Sci. 2021;4(2):424–435. 33860172 10.1021/acsptsci.1c00014 PMC8033773

[40] Muthukumaraswamy SD, Forsyth A, Lumley T. Blinding and expectancy confounds in psychedelic randomized controlled trials. Expert Rev Clin Pharmacol. 2021;14(9):1133–1152. 34038314 10.1080/17512433.2021.1933434

[41] Gukasyan N, Nayak SM. Psychedelics, placebo effects, and set and setting: insights from common factors theory of psychotherapy. Transcult Psychiatry. 2022;59(5):652–664. 33499762 10.1177/1363461520983684

[42] Jylkka J. Reconciling mystical experiences with naturalistic psychedelic science: reply to Sanders and Zijlmans. ACS Pharmacol Transl Sci. 2021;4(4):1468–1470. 34423278 10.1021/acsptsci.1c00137 PMC8369668

[43] Lii TR, Smith AE, Flohr JR, et al. Randomized trial of ketamine masked by surgical anesthesia in patients with depression. Nat Mental Health. 2023;1(11):876–886. 38188539 10.1038/s44220-023-00140-x PMC10769130

[44] Butler M, Jelen L, Rucker J. Expectancy in placebo-controlled trials of psychedelics: if so, so what? Psychopharmacology. 2022;239(10):3047–3055. 36063208 10.1007/s00213-022-06221-6 PMC9481484

[45] Hartogsohn I. Constructing drug effects: a history of set and setting. Drug Sci Policy Law. 2017;3(0):1–17.

[46] Uthaug M, Mason N, Toennes SW, et al. A placebo-controlled study of the effects of ayahuasca, set and setting on mental health of participants in ayahuasca group retreats. Psychopharmacology. 2021;238:1899–1910. 33694031 10.1007/s00213-021-05817-8 PMC8233273

[47] Smith WR, Sisti D. Ethics and ego dissolution: the case of psilocybin. J Med Ethics. 2021;47(12):807–814. 10.1136/medethics-2020-106070 PMC9202314 32461241

[48] da Costa SC, Sofuoglu M. On the edges: the ethics of human studies with psychedelic substances. *Ethics and clinical neuroinnovation: fundamentals, stakeholders, case studies, and emerging issues.* Springer; 2023:153–171.

[49] Harrison TR. Altered stakes: identifying gaps in the informed consent process for psychedelic-assisted therapy trials. J Psychedelic Stud. 2023;7(1):48–60.

[50] Brennan W, Jackson MA, MacLean K, Ponterotto JG. A qualitative exploration of relational ethical challenges and practices in psychedelic healing. J Humanist Psychol. 2021:1–31.

[51] Gilbert J. Contesting consent in sex education. Sex Edu. 2018;18(3):268–279.

[52] Cahill AJ. Recognition, desire, and unjust sex. Hypatia. 2014;29(2):303–319.

[53] Wright J. Trauma-informed consent education: understanding the grey area of consent through the experiences of youth trauma survivors. Atlantis. 2022;43(1):19–31.

[54] Lin Y-K, Liu K-T, Chen C-W, et al. How to effectively obtain informed consent in trauma patients: a systematic review. BMC Med Ethics. 2019;20(8):1–15. 30674301 10.1186/s12910-019-0347-0 PMC6343333

[55] Villiger D, Trachsel M. With great power comes great vulnerability: an ethical analysis of psychedelics’ therapeutic mechanisms proposed by the REBUS hypothesis. J Med Ethics. 2023;49(12):826–832. 37045591 10.1136/jme-2022-108816

[56] Sanders JW, Zijlmans J. Moving past mysticism in psychedelic science. ACS Pharmacol Transl Sci. 2021;4(3):1253–1255. 34151217 10.1021/acsptsci.1c00097 PMC8205234

[57] Breeksema JJ, Van Elk M. Working with weirdness: a response to “moving past mysticism in psychedelic science”. ACS Pharmacol Transl Sci. 2021;4(4):1471–1474. 34423279 10.1021/acsptsci.1c00149 PMC8369678

[58] Letheby C, Mattu J. Philosophy and classic psychedelics: a review of some emerging themes. J Psychedelic Stud. 2022;5(3):166–175.

[59] Gerber K, Flores IG, Ruiz AC, Ali I, Ginsberg NL, Schenberg EE. Ethical concerns about psilocybin intellectual property. ACS Pharmacol Transl Sci. 2021;4(2):573–577. 33860186 10.1021/acsptsci.0c00171 PMC8033603

[60] Dos Santos RG, Bouso JC, Rocha JM, Rossi GN, Hallak JE. The use of classic hallucinogens/psychedelics in a therapeutic context: healthcare policy opportunities and challenges. Risk Manag Healthc Policy. 2021;14:901–910. 33707976 10.2147/RMHP.S300656 PMC7943545

[61] Williams K, Romero OSG, Braunstein M, Brant S. Indigenous Philosophies and the" Psychedelic Renaissance". Anthropol Consciousness. 2022;33(2):506–527.

[62] Ponomarenko P, Seragnoli F, Calder A, Oehen P, Hasler G. Can psychedelics enhance group psychotherapy? A discussion on the therapeutic factors. J Psychopharmacol. 2023;37(7):660–678. 36855289 10.1177/02698811231155117 PMC10350738

[63] Schlag AK, Hindocha C, Zafar R, Nutt DJ, Curran HV. Cannabis based medicines and cannabis dependence: a critical review of issues and evidence. J Psychopharmacol. 2021;35(7):773–785. 33593117 10.1177/0269881120986393 PMC8278552

[64] Bonini SA, Premoli M, Tambaro S, et al. Cannabis sativa: a comprehensive ethnopharmacological review of a medicinal plant with a long history. J Ethnopharmacol. 2018;227:300–315. 30205181 10.1016/j.jep.2018.09.004

[65] Charitos IA, Gagliano-Candela R, Santacroce L, Bottalico L. The Cannabis spread throughout the continents and its therapeutic use in history. Endocr Metab Immune Disord Drug Targets. 2021;21(3):407–417. 32433013 10.2174/1871530320666200520095900

[66] Warf B. High points: an historical geography of cannabis. Geogr Rev. 2014;104(4):414–438.

[67] Mills JH. Cannabis Britannica: empire, trade, and prohibition 1800–1928. OUP Oxford; 2003.

[68] Sheehan BE, Grucza RA, Plunk AD. Association of racial disparity of cannabis possession arrests among adults and youths with statewide cannabis decriminalization and legalization. American Medical Association; 2021:e213435. 10.1001/jamahealthforum.2021.3435 PMC8727041 35977162

[69] Decorte T, Lenton S, Wilkins C. Legalizing cannabis: experiences, lessons and scenarios. Routledge; 2020.

[70] Owusu-Bempah A, Luscombe A. Race, cannabis and the Canadian war on drugs: an examination of cannabis arrest data by race in five cities. Int J Drug Policy. 2021;91:102937. 10.1016/j.drugpo.2020.102937 33011019

[71] Møller K. Policy displacement and disparate sanctioning from policing cannabis in Denmark. J Scan Stud Criminol Crime Prev. 2010;11(2):135–150.

[72] Theodore R, Ratima M, Potiki T, Boden J, Poulton R. Cannabis, the cannabis referendum and Māori youth: a review from a lifecourse perspective. Kōtuitui: NZ J Social Sci Online. 2021;16(1):1–17.

[73] Hernández Castillo RA. Racialized geographies and the “war on drugs”: gender violence, militarization, and criminalization of Indigenous peoples. J Latin Am Caribb Anthropol. 2019;24(3):635–652.

[74] Baker J, Goh D. The cannabis cautioning scheme three years on: an implementation and outcome evaluation. NSW Bureau of Crime Statistics and Research Sydney; 2004.

[75] Wheeldon J, Heidt J. Cannabis, research ethics, and a duty of care. Res Ethics. 2023;19(3):250–287.

[76] ElSohly MA, Slade D. Chemical constituents of marijuana: the complex mixture of natural cannabinoids. Life Sci. 2005;78(5):539–548. 16199061 10.1016/j.lfs.2005.09.011

[77] Ladha KS, Ajrawat P, Yang Y, Clarke H. Understanding the medical chemistry of the cannabis plant is critical to guiding real world clinical evidence. Molecules. 2020;25(18):4042. 32899678 10.3390/molecules25184042 PMC7570835

[78] Shahbazi F, Grandi V, Banerjee A, Trant JF. Cannabinoids and cannabinoid receptors: the story so far. IScience. 2020;23(7):101301. 10.1016/j.isci.2020.101301 PMC7339067 32629422

[79] Bonn-Miller MO, Pollack Jr CV, Casarett D, et al. Priority considerations for medicinal cannabis-related research. Cannabis Cannabinoid Res. 2019;4(3):139–157. 31579832 10.1089/can.2019.0045 PMC6757233

[80] Lewis-Bakker MM, Yang Y, Vyawahare R, Kotra LP. Extractions of medical cannabis cultivars and the role of decarboxylation in optimal receptor responses. Cannabis Cannabinoid Res. 2019;4(3):183–194. 31559334 10.1089/can.2018.0067 PMC6757234

[81] Vandrey R, Raber JC, Raber ME, Douglass B, Miller C, Bonn-Miller MO. Cannabinoid dose and label accuracy in edible medical cannabis products. Jama. 2015;313(24):2491–2493. 26103034 10.1001/jama.2015.6613

[82] MacCallum CA, Lo LA, Pistawka CA, Boivin M. A clinical framework for evaluating cannabis product quality and safety. Cannabis Cannabinoid Res. 2023;8(3):567–574. 35049330 10.1089/can.2021.0137 PMC10249738

[83] Yang Y, Vyawahare R, Lewis-Bakker M, Clarke HA, Wong AH, Kotra LP. Bioactive chemical composition of Cannabis extracts and cannabinoid receptors. Molecules. 2020;25(15):3466. 32751516 10.3390/molecules25153466 PMC7436063

[84] Lintzeris N, Mills L, Suraev A, et al. Medical cannabis use in the Australian community following introduction of legal access: the 2018–2019 Online Cross-Sectional Cannabis as Medicine Survey (CAMS-18). Harm Reduct J. 2020;17(37):1–12. 32513180 10.1186/s12954-020-00377-0 PMC7278204

[85] Shiplo S, Asbridge M, Leatherdale ST, Hammond D. Medical cannabis use in Canada: vapourization and modes of delivery. Harm Reduct J. 2016;13(30):1–10. 27793174 10.1186/s12954-016-0119-9 PMC5086046

[86] Lopera V, Rodríguez A, Amariles P. Clinical relevance of drug interactions with cannabis: a systematic review. J Clin Med. 2022;11(5):1154. 35268245 10.3390/jcm11051154 PMC8911401

[87] Balachandran P, Elsohly M, Hill KP. Cannabidiol interactions with medications, illicit substances, and alcohol: a comprehensive review. J Gen Intern Med. 2021;36(7):2074–2084. 33515191 10.1007/s11606-020-06504-8 PMC8298645

[88] Devinsky O, Marsh E, Friedman D, et al. Cannabidiol in patients with treatment-resistant epilepsy: an open-label interventional trial. Lancet Neurol. 2016;15(3):270–278. 26724101 10.1016/S1474-4422(15)00379-8

[89] Anderson LL, Doohan PT, Oldfield L, et al. Citalopram and cannabidiol: in vitro and in vivo evidence of pharmacokinetic interactions relevant to the treatment of anxiety disorders in young people. J Clin Psychopharmacol. 2021;41(5):525–533. 34121064 10.1097/JCP.0000000000001427

[90] Rey AA, Purrio M, Viveros M-P, Lutz B. Biphasic effects of cannabinoids in anxiety responses: CB1 and GABAB receptors in the balance of GABAergic and glutamatergic neurotransmission. Neuropsychopharmacology. 2012;37(12):2624–2634. 22850737 10.1038/npp.2012.123 PMC3473327

[91] Pratt M, Stevens A, Thuku M, et al. Benefits and harms of medical cannabis: a scoping review of systematic reviews. Syst Rev. 2019;8(320):1–35. 31823819 10.1186/s13643-019-1243-x PMC6905063

[92] Solmi M, De Toffol M, Kim JY, et al. Balancing risks and benefits of cannabis use: umbrella review of meta-analyses of randomised controlled trials and observational studies. Bmj. 2023;382:e072348. 37648266 10.1136/bmj-2022-072348 PMC10466434

[93] Doeve BH, Van De Meeberg MM, Van Schaik FD, Fidder HH. A systematic review with meta-analysis of the efficacy of cannabis and cannabinoids for inflammatory bowel disease: what can we learn from randomized and nonrandomized studies? J Clin Gastroenterol. 2021;55(9):798–809. 32675631 10.1097/MCG.0000000000001393

[94] Oikonomou P, Jost W. Randomized controlled trials on the use of cannabis-based medicines in movement disorders: a systematic review. J Neural Transm. 2022;129(10):1247–1256. 35859051 10.1007/s00702-022-02529-x

[95] MacCallum CA, Russo EB. Practical considerations in medical cannabis administration and dosing. Eur J Intern Med. 2018;49:12–19. 29307505 10.1016/j.ejim.2018.01.004

[96] Lim K, See YM, Lee J. A systematic review of the effectiveness of medical cannabis for psychiatric, movement and neurodegenerative disorders. Clin Psychopharmacol Neurosci. 2017;15(4):301. 29073741 10.9758/cpn.2017.15.4.301 PMC5678490

[97] Shakya DR, Upadhaya SR, Neupane H, Subedi R. Considerations for the use of medical cannabis: an overview of benefits and harms. Biomed J Sci & Tech Res. 2021;36(4):28746–53.

[98] Montero-Oleas N, Arevalo-Rodriguez I, Nuñez-González S, Viteri-García A, Simancas-Racines D. Therapeutic use of cannabis and cannabinoids: an evidence mapping and appraisal of systematic reviews. BMC Complement Med Ther. 2020;20:1–15. 32020875 10.1186/s12906-019-2803-2 PMC7076827

[99] Orsolini L, Chiappini S, Volpe U, et al. Use of medicinal cannabis and synthetic cannabinoids in post-traumatic stress disorder (PTSD): a systematic review. Medicina. 2019;55(9):525. 31450833 10.3390/medicina55090525 PMC6780141

[100] Black N, Stockings E, Campbell G, et al. Cannabinoids for the treatment of mental disorders and symptoms of mental disorders: a systematic review and meta-analysis. Lancet Psychiatry. 2019;6(12):995–1010. 31672337 10.1016/S2215-0366(19)30401-8 PMC6949116

[101] Hoch E, Niemann D, von Keller R, et al. How effective and safe is medical cannabis as a treatment of mental disorders? A systematic review. Eur Arch Psychiatry Clin Neurosci. 2019;269:87–105. 30706168 10.1007/s00406-019-00984-4 PMC6595000

[102] Medicinal Cannabis: special access scheme category-b applications database. 1 May 2024. Accessed 4 August 2024. https://dashboard-data.health.gov.au/single/?appid=1066afbe-2b37-427d-8c47-2caa5082cccc&sheet=088f611b-10de-4d72-be68-ccf8d12c54e9&select=clearall

[103] Johnston KJ, Huckins LM. Chronic pain and psychiatric conditions. Complex Psychiatry. 2023;9(1–4):24–43. 37034825 10.1159/000527041 PMC10080192

[104] Cairns EA, Benson MJ, Bedoya-Pérez MA, et al. Medicinal cannabis for psychiatry-related conditions: an overview of current Australian prescribing. Front Pharmacol. 2023;14:1142680. 10.3389/fphar.2023.1142680 PMC10279775 37346297

[105] Ragnhildstveit A, Kaiyo M, Snyder MB, et al. Cannabis-assisted psychotherapy for complex dissociative posttraumatic stress disorder: a case report. Front PSychiatry. 2023;14:1051542. 10.3389/fpsyt.2023.1051542 PMC9947284 36846226

[106] Earleywine M, Ueno LF, Mian MN, Altman BR. Cannabis-induced oceanic boundlessness. J Psychopharmacol. 2021;35(7):841–847. 33779383 10.1177/0269881121997099

[107] Kayser RR, Raskin M, Snorrason I, Hezel DM, Haney M, Simpson HB. Cannabinoid augmentation of exposure-based psychotherapy for obsessive-compulsive disorder. J Clin Psychopharmacol. 2020;40(2):207–210. 32068563 10.1097/JCP.0000000000001179 PMC7206660

[108] Spinella TC, Stewart SH, Barrett SP. Context matters: characteristics of solitary versus social cannabis use. Drug Alcohol Rev. 2019;38(3):316–320. 30779237 10.1111/dar.12912

[109] Schlossarek S, Kempkensteffen J, Reimer J, Verthein U. Psychosocial determinants of cannabis dependence: a systematic review of the literature. Eur Addict Res. 2016;22(3):131–144. 26551358 10.1159/000441777

[110] Karanges EA, Suraev A, Elias N, Manocha R, McGregor IS. Knowledge and attitudes of Australian general practitioners towards medicinal cannabis: a cross-sectional survey. BMJ Open. 2018;8(7):e022101. 10.1136/bmjopen-2018-022101 PMC6042562 29970456

[111] Rønne ST, Rosenbæk F, Pedersen LB, et al. Physicians’ experiences, attitudes, and beliefs towards medical cannabis: a systematic literature review. BMC Family Pract. 2021;22:1–21. 10.1186/s12875-021-01559-w PMC8532330 34674661

[112] Gardiner KM, Singleton JA, Sheridan J, Kyle GJ, Nissen LM. Health professional beliefs, knowledge, and concerns surrounding medicinal cannabis–a systematic review. PLoS One. 2019;14(5):e0216556. 31059531 10.1371/journal.pone.0216556 PMC6502454

[113] Glickman A, Sisti D. Prescribing medical cannabis: ethical considerations for primary care providers. J Med Ethics. 2020;46(4):227–230. 31852743 10.1136/medethics-2019-105759

[114] Van Rensburg R, Pillay-Fuentes Lorente V, Blockman M, Moodley K, Wilmshurst J, Decloedt E. Medical cannabis: what practitioners need to know. S Afr Med J. 2020;110(3):192–196. 32657695 10.7196/SAMJ.2020.v110i3.14403

[115] Martin JH, Bonomo Y, Reynolds AD. Compassion and evidence in prescribing cannabinoids: a perspective from the Royal Australasian College of Physicians. Med J Aus. 2018;208(3):107–109. 10.5694/mja17.01004 29438641

[116] Lopez-Quintero C, de los Cobos JP, Hasin DS, et al. Probability and predictors of transition from first use to dependence on nicotine, alcohol, cannabis, and cocaine: results of the National Epidemiologic Survey on Alcohol and Related Conditions (NESARC). Drug Alcohol Depend. 2011;115(1–2):120–130. 21145178 10.1016/j.drugalcdep.2010.11.004 PMC3069146

[117] Feingold D, Goor-Aryeh I, Bril S, Delayahu Y, Lev-Ran S. Problematic use of prescription opioids and medicinal cannabis among patients suffering from chronic pain. Pain Med. 2017;18(2):294–306. 28204792 10.1093/pm/pnw134

[118] Boehnke KF, Scott JR, Litinas E, Sisley S, Williams DA, Clauw DJ. High-frequency medical cannabis use is associated with worse pain among individuals with chronic pain. J Pain. 2020;21(5–6):570–581. 31560957 10.1016/j.jpain.2019.09.006 PMC7089844

[119] Montgomery BW, Allen J. Cannabis policy in the 21st century: mandating an equitable future and shedding the racist past. Clin Ther. 2023;45(6):541–550. 37414505 10.1016/j.clinthera.2023.05.001

[120] Orenstein DG, Glantz SA. Regulating cannabis manufacturing: applying public health best practices from tobacco control. J Psychoactive Drugs. 2018;50(1):19–32. 29438634 10.1080/02791072.2017.1422816 PMC5878137

[121] Moodie R, Bennett E, Kwong EJL, et al. Ultra-processed profits: the political economy of countering the global spread of ultra-processed foods–a synthesis review on the market and political practices of transnational food corporations and strategic public health responses. Int J Health Policy Manag. 2021;10(12):968. 34124866 10.34172/ijhpm.2021.45 PMC9309965

[122] Hoe C, Weiger C, Minosa MKR, Alonso F, Koon AD, Cohen JE. Strategies to expand corporate autonomy by the tobacco, alcohol and sugar-sweetened beverage industry: a scoping review of reviews. Global Health. 2022;18(1):17. 35164801 10.1186/s12992-022-00811-x PMC8845406

[123] Mialon M. An overview of the commercial determinants of health. Global Health. 2020;16:1–7. 32807183 10.1186/s12992-020-00607-x PMC7433173

[124] Alibudbud R. Google Trends for health research: its advantages, application, methodological considerations, and limitations in psychiatric and mental health infodemiology. Front Big Data. 2023;6:1132764. 10.3389/fdata.2023.1132764 PMC10083382 37050919

[125] Bierut T, Krauss MJ, Sowles SJ, Cavazos-Rehg PA. Exploring marijuana advertising on Weedmaps, a popular online directory. Prev Sci. 2017;18:183–192. 27534665 10.1007/s11121-016-0702-z PMC5247325

[126] Rup J, Goodman S, Hammond D. Cannabis advertising, promotion and branding: differences in consumer exposure between ‘legal’and ‘illegal’markets in Canada and the US. Prev Med. 2020;133:106013. 10.1016/j.ypmed.2020.106013 32027914

[127] Sheikhan NY, Pinto AM, Nowak DA, et al. Compliance with cannabis act regulations regarding online promotion among Canadian commercial cannabis-licensed firms. JAMA Netw Open. 2021;4(7):e2116551. 34251442 10.1001/jamanetworkopen.2021.16551 PMC8276085

[128] MacPhail SL, Bedoya-Pérez MA, Cohen R, Kotsirilos V, McGregor IS, Cairns EA. Medicinal cannabis prescribing in Australia: an analysis of trends over the first five years. Front Pharmacol. 2022;13:1368. 10.3389/fphar.2022.885655 PMC9127064 35620292

[129] Graham M, Chiu V, Stjepanović D, Hall W. A provisional evaluation of Australia’s medical cannabis program. Int J Drug Policy. 2023;122:104210. 37813082 10.1016/j.drugpo.2023.104210

[130] Hallinan CM, Eden E, Graham M, et al. Over the counter low-dose cannabidiol: a viewpoint from the ACRE Capacity Building Group. J Psychopharmacol. 2022;36(6):661–665. 34344208 10.1177/02698811211035394

[131] Gunning M, Illes J. Coverage of medical cannabis by Canadian news media: ethics, access, and policy. Int J Drug Policy. 2021;97:103361. 10.1016/j.drugpo.2021.103361 34252784

[132] Liebling JP, Clarkson NJ, Gibbs BW, Yates AS, O’Sullivan SE. An analysis of over-the-counter cannabidiol products in the United Kingdom. Cannabis Cannabinoid Res. 2022;7(2):207–213. 33998849 10.1089/can.2019.0078 PMC9070743

[133] Lhooq M. Lab Tests From Diamond Shruumz Mushroom Brand Reveals Deceptive Practices Double Blind; 1 July 2024. Accessed 14 September 2024. https://doubleblindmag.com/fda-investigates-diamond-shruumz/

[134] Worthington E. Several hospitalised after consuming Uncle Frog mushroom gummies, sparking public health warning and national recall. *ABC News.* Australian Broadcasting Corporation; 27 June 2024. Accessed 14 September 2024. https://www.abc.net.au/news/2024-06-27/uncle-frog-mushroom-gummies-public-health-warning/104028454

[135] Ilan Y. Digital medical cannabis as market differentiator: second-generation artificial intelligence systems to improve response. Front Med. 2022;8:788777. 10.3389/fmed.2021.788777 PMC8818992 35141242

[136] Hallinan CM, Khademi Habibabadi S, Conway M, Bonomo YA. Social media discourse and internet search queries on cannabis as a medicine: a systematic scoping review. PloS One. 2023;18(1):e0269143. 36662832 10.1371/journal.pone.0269143 PMC9858862

[137] Masterson D, Areskoug Josefsson K, Robert G, Nylander E, Kjellström S. Mapping definitions of co-production and co-design in health and social care: a systematic scoping review providing lessons for the future. Health Expect. 2022;25(3):902–913. 35322510 10.1111/hex.13470 PMC9122425

[138] Tanaka K, Mayne L, Khalil A, et al. The role of the endocannabinoid system in aetiopathogenesis of endometriosis: a potential therapeutic target. Eur J Obstetr Gynecol Reproduct Biol. 2020;244:87–94. 10.1016/j.ejogrb.2019.11.012 31785471

[139] Hunt G, Antin TM, Frank VA. Routledge handbook of intoxicants and intoxication. Taylor & Francis; 2023.

[140] Rasmussen N. America’s first amphetamine epidemic 1929–1971: a quantitative and qualitative retrospective with implications for the present. Am J Public Health. 2008;98(6):974–985. 18445805 10.2105/AJPH.2007.110593 PMC2377281

[141] Cohen PA, Goday A, Swann JP. The return of rainbow diet pills. Am J Public Health. 2012;102(9):1676–1686. 22813089 10.2105/AJPH.2012.300655 PMC3482033

[142] Vekaria V, Bose B, Murphy SM, Avery J, Alexopoulos G, Pathak J. Association of co-occurring opioid or other substance use disorders with increased healthcare utilization in patients with depression. Transl Psychiatry. 2021;11(1):265. 33941761 10.1038/s41398-021-01372-0 PMC8093211

[143] Preuss UW, Schaefer M, Born C, Grunze H. Bipolar disorder and comorbid use of illicit substances. Medicina. 2021;57(11):1256. 34833474 10.3390/medicina57111256 PMC8623998

[144] Lähteenvuo M, Batalla A, Luykx JJ, et al. Morbidity and mortality in schizophrenia with comorbid substance use disorders. Acta Psychiatr Scand. 2021;144(1):42–49. 33650123 10.1111/acps.13291 PMC8359349

[145] Chan WW, Shum KKm, Sonuga-Barke EJ. Attention-deficit/hyperactivity disorder (ADHD) in cultural context: do parents in Hong Kong and the United Kingdom adopt different thresholds when rating symptoms, and if so why? Int J Methods Psychiatric Res. 2022;31(3):e1923. 10.1002/mpr.1923 PMC9464328 35670761

[146] Watabe Y, Lee SK, Matsuhashi Y. Cross-cultural investigation in differential perceptions of externalizing symptoms. Jpn PsycholRes. 2024;66(3):255–263.

[147] Graf WD, Miller G, Nagel SK. Addressing the problem of ADHD medication as neuroenhancements. Expert Rev Neurother. 2014;14(5):569–581. 24738763 10.1586/14737175.2014.908707

[148] Sakakibara E. The polysemy of psychotropic drugs: continuity and overlap between neuroenhancement, treatment, prevention, pain relief, and pleasure-seeking in a clinical setting. BMC Med Ethics. 2020;21:1–8. 32631307 10.1186/s12910-020-00497-z PMC7336425

[149] Rosenblat JD, Simon GE, Sachs GS, et al. Treatment effectiveness and tolerability outcomes that are most important to individuals with bipolar and unipolar depression. J Affect Disord. 2019;243:116–120. 30241026 10.1016/j.jad.2018.09.027

[150] Forlini C, Hall W. The is and ought of the ethics of neuroenhancement: mind the gap. Front Psychol. 2016;6:1998. 10.3389/fpsyg.2015.01998 PMC4705235 26779100

[151] Ilieva IP, Farah MJ. Enhancement stimulants: perceived motivational and cognitive advantages. Front Neurosci. 2013;7:198. 24198755 10.3389/fnins.2013.00198 PMC3813924

[152] Ricci G. Pharmacological human enhancement: an overview of the looming bioethical and regulatory challenges. Front Psychiatry. 2020;11:53. 32127792 10.3389/fpsyt.2020.00053 PMC7037021

[153] Schleim S, Quednow BB. Debunking the ethical neuroenhancement debate. *Rethinking cognitive enhancement.* 2017:164–176.

[154] Franke AG, Lieb K. Pharmacological neuroenhancement and brain doping: chances and risks. Bundesgesundheitsblatt-Gesundheitsforschung-Gesundheitsschutz. 2010;53:853–860. 20700786 10.1007/s00103-010-1105-0

[155] Normann C, Boldt J, Maio G, Berger M. Options, limits and ethics of pharmacological neuroenhancement. Der Nervenarzt. 2010;81:66–74. 19851745 10.1007/s00115-009-2858-2

[156] Kortekaas-Rijlaarsdam AF, Luman M, Sonuga-Barke E, Oosterlaan J. Does methylphenidate improve academic performance? A systematic review and meta-analysis. Eur Child Adolescent Psychiatry. 2019;28:155–164. 10.1007/s00787-018-1106-3 29353323

[157] Advokat C. What are the cognitive effects of stimulant medications? Emphasis on adults with attention-deficit/hyperactivity disorder (ADHD). Neurosci Biobehav Rev. 2010;34(8):1256–1266. 20381522 10.1016/j.neubiorev.2010.03.006

[158] Farrell M, Martin NK, Stockings E, et al. Responding to global stimulant use: challenges and opportunities. The Lancet. 2019;394(10209):1652–1667. 10.1016/S0140-6736(19)32230-5 PMC6924572 31668409

[159] Asherson P. Drug treatments for ADHD reduce risk of substance use disorders. Am Psychiatric Assoc; 2017. p. 827–828. 10.1176/appi.ajp.2017.17070733 28859502

[160] Bastos LS. Psychotic outcomes following amphetamine use: a Danish National Cohort Study. Schizophrenia Bulletin. 2017;43(6):1280–1290.28586480

[161] Fleming T, Barker A, Ivsins A, Vakharia S, McNeil R. Stimulant safe supply: a potential opportunity to respond to the overdose epidemic. Harm Reduct J. 2020;17(1):6. 31924209 10.1186/s12954-019-0351-1 PMC6954588

[162] Siefried KJ, Acheson LS, Lintzeris N, Ezard N. Pharmacological treatment of methamphetamine/amphetamine dependence: a systematic review. CNS Drugs. 2020;34(4):337–365. 32185696 10.1007/s40263-020-00711-x PMC7125061

[163] Woon L, Hazli Z, Gan L. Pharmacotherapy for comorbid adult attention-deficit hyperactivity disorder and stimulant dependence: a systematic review. IIUM Med J Malaysia. 2018;17(2)

[164] *World Drug Report* . 2024 Accessed 14 September, 2024. https://www.unodc.org/unodc/en/data-and-analysis/world-drug-report-2024.html

[165] Barbuti M, Maiello M, Spera V, et al. Challenges of treating ADHD with comorbid substance use disorder: considerations for the clinician. J Clin Med. 2023;12(9):3096. 37176536 10.3390/jcm12093096 PMC10179386

[166] Tomažič T, Čelofiga AK. Ethical aspects of the abuse of pharmaceutical enhancements by healthy people in the context of improving cognitive functions. Philos Ethics Humanit Med. 2019;14:1–6. 31023334 10.1186/s13010-019-0076-5 PMC6482530

[167] Bavarian N, Flay BR, Ketcham PL, Smit E. The illicit use of prescription stimulants on college campuses: a theory-guided systematic review. Health Educ Behav. 2015;42(6):719–729. 26032000 10.1177/1090198115580576 PMC6553472

[168] Faraone SV, Rostain AL, Montano CB, Mason O, Antshel KM, Newcorn JH. Systematic review: nonmedical use of prescription stimulants: risk factors, outcomes, and risk reduction strategies. J Am Acad Child Adolesc Psychiatry. 2020;59(1):100–112. 31326580 10.1016/j.jaac.2019.06.012

[169] Sadek J. Malingering and stimulant medications abuse, misuse and diversion. Brain Sci. 2022;12(8):1004. 36009067 10.3390/brainsci12081004 PMC9406161

[170] Lam C, Figueroa W, Yomogida K, Bavarian N. Prescription stimulant diversion on a college campus: intrapersonal, interpersonal, and environmental correlates. J Drug Issues. 2020;50(3):329–340. 34305170 10.1177/0022042620917103 PMC8297922

[171] Compton WM, Han B, Blanco C, Johnson K, Jones CM. Prevalence and correlates of prescription stimulant use, misuse, use disorders, and motivations for misuse among adults in the United States. Am J Psychiatry. 2018;175(8):741–755. 29656665 10.1176/appi.ajp.2018.17091048 PMC6070393

[172] Pham T, Milanaik R, Kaplan A, Papaioannou H, Adesman A. Household diversion of prescription stimulants: medication misuse by parents of children with attention-deficit/hyperactivity disorder. J Child and Adolesc Psychopharmacol. 2017;27(8):741–746. 28686059 10.1089/cap.2016.0058

[173] Penley B, Chen H-H, Eckel SF, Ozawa S. Characteristics of online pharmacies selling Adderall. J Am Pharm Assoc. 2021;61(1):e103–e109. 10.1016/j.japh.2020.07.022 PMC7476499 32912756

[174] Jillani Z, Reinhard L, Hertig J. A narrative review of illegal online pharmacies and contemporary issues with restricting FDA-approved medication access. J Med Access. 2023;7:27550834231220512. 10.1177/27550834231220512 PMC10750516 38149839

[175] Boland H, DiSalvo M, Fried R, et al. A literature review and meta-analysis on the effects of ADHD medications on functional outcomes. J Psychiatr Res. 2020;123:21–30. 32014701 10.1016/j.jpsychires.2020.01.006

[176] Andersen SL, Navalta CP. Altering the course of neurodevelopment: a framework for understanding the enduring effects of psychotropic drugs. Int J Dev Neurosci. 2004;22(5–6):423–440. 15380841 10.1016/j.ijdevneu.2004.06.002

[177] Schrantee A, Bouziane C, Bron E, et al. Long-term effects of stimulant exposure on cerebral blood flow response to methylphenidate and behavior in attention-deficit hyperactivity disorder. Brain Imaging Behav. 2018;12:402–410. 28321605 10.1007/s11682-017-9707-x PMC5880865

[178] Loureiro-Vieira S, Costa VM, de Lourdes Bastos M, Carvalho F, Capela JP. Methylphenidate effects in the young brain: friend or foe? Int J Dev Neurosci. 2017;60:34–47. 28412445 10.1016/j.ijdevneu.2017.04.002

[179] Bottelier MA, Schouw ML, Klomp A, et al. The effects of Psychotropic drugs On Developing brain (ePOD) study: methods and design. BMC Psychiatry. 2014;14:1–12. 10.1186/1471-244X-14-48 PMC3930821 24552282

[180] Catalá-López F, Hutton B, Núñez-Beltrán A, et al. The pharmacological and non-pharmacological treatment of attention deficit hyperactivity disorder in children and adolescents: a systematic review with network meta-analyses of randomised trials. PloS One. 2017;12(7):e0180355. 28700715 10.1371/journal.pone.0180355 PMC5507500

[181] De Crescenzo F, Cortese S, Adamo N, Janiri L. Pharmacological and non-pharmacological treatment of adults with ADHD: a meta-review. BMJ Ment Health. 2017;20(1):4–11. 10.1136/eb-2016-102415 PMC10699262 27993933

[182] Cortese S, Coghill D. Twenty years of research on attention-deficit/hyperactivity disorder (ADHD): looking back, looking forward. BMJ Ment Health. 2018;21(4):173–176. 10.1136/ebmental-2018-300050 PMC10270437 30301823

[183] Ringer N. Living with ADHD: a meta-synthesis review of qualitative research on children’s experiences and understanding of their ADHD. Int J Disability Dev Educ. 2020;67(2):208–224.

[184] Druedahl LC, Kälvemark Sporrong S. Managing complexity: exploring decision making on medication by young adults with ADHD. Pharmacy. 2018;6(2):33. 29671768 10.3390/pharmacy6020033 PMC6025481

[185] Rashid MA, Lovick S, Llanwarne NR. Medication-taking experiences in attention deficit hyperactivity disorder: a systematic review. Family Practice. 2018;35(2):142–150. 28973393 10.1093/fampra/cmx088 PMC5892172

[186] Jacobs D. Experiences of children and adolescents with attention-deficit/hyperactivity disorder taking methylphenidate. Dev Med Child Neurol. 2023;65(12):1587–1595. 37154566 10.1111/dmcn.15636

[187] Castells X, Saez M, Barcheni M, et al. Placebo response and its predictors in Attention Deficit Hyperactivity Disorder: a meta-analysis and comparison of meta-regression and MetaForest. Int J Neuropsychopharmacol. 2022;25(1):26–35. 34355753 10.1093/ijnp/pyab054 PMC8756096

[188] Schweren L, Hoekstra P, van Lieshout M, et al. Long-term effects of stimulant treatment on ADHD symptoms, social–emotional functioning, and cognition. Psychol Med. 2019;49(2):217–223. 29530108 10.1017/S0033291718000545

[189] Janssen T, Hillebrand A, Gouw A, et al. Neural network topology in ADHD; Evidence for maturational delay and default-mode network alterations. Clin Neurophysiol. 2017;128(11):2258–2267. 29028500 10.1016/j.clinph.2017.09.004

[190] Hyman SE. Might stimulant drugs support moral agency in ADHD children? J Med Ethics. 2013;39(6):369–370. 23001921 10.1136/medethics-2012-100846 PMC3664373

